# Interleukin-18 Amplifies Macrophage Polarization and Morphological Alteration, Leading to Excessive Angiogenesis

**DOI:** 10.3389/fimmu.2018.00334

**Published:** 2018-03-06

**Authors:** Takuro Kobori, Shinichi Hamasaki, Atsuhiro Kitaura, Yui Yamazaki, Takashi Nishinaka, Atsuko Niwa, Shinichi Nakao, Hidenori Wake, Shuji Mori, Tadashi Yoshino, Masahiro Nishibori, Hideo Takahashi

**Affiliations:** ^1^Department of Pharmacology, Faculty of Medicine, Kindai University, Osaka-Sayama, Japan; ^2^Department of Anesthesiology, Faculty of Medicine, Kindai University, Osaka-Sayama, Japan; ^3^Department of Pharmacology, Graduate School of Medicine, Dentistry and Pharmaceutical Sciences, Okayama University, Okayama, Japan; ^4^Department of Pharmacology, School of Pharmacy, Shujitsu University, Okayama, Japan; ^5^Department of Pathology, Graduate School of Medicine, Dentistry and Pharmaceutical Sciences, Okayama University, Okayama, Japan

**Keywords:** macrophage, CD163, angiogenesis, interleukin-18, osteopontin, thrombin

## Abstract

M2 macrophage (Mφ) promotes pathologic angiogenesis through a release of pro-angiogenic mediators or the direct cell–cell interaction with endothelium in the micromilieu of several chronic inflammatory diseases, including rheumatoid arthritis and cancer, where interleukin (IL)-18 also contributes to excessive angiogenesis. However, the detailed mechanism remains unclear. The aim of this study is to investigate the mechanism by which M2 Mφs in the micromilieu containing IL-18 induce excessive angiogenesis in the *in vitro* experimental model using mouse Mφ-like cell line, RAW264.7 cells, and mouse endothelial cell line, b.End5 cells. We discovered that IL-18 acts synergistically with IL-10 to amplify the production of Mφ-derived mediators like osteopontin (OPN) and thrombin, yielding thrombin-cleaved form of OPN generation, which acts through integrins α4/α9, thereby augmenting M2 polarization of Mφ with characteristics of increasing surface CD163 expression in association with morphological alteration. Furthermore, the results of visualizing temporal behavior and morphological alteration of Mφs during angiogenesis demonstrated that M2-like Mφs induced excessive angiogenesis through the direct cell–cell interaction with endothelial cells, possibly mediated by CD163.

## Introduction

Macrophages (Mφs) play essential roles in tissue homeostasis and immunity (1), and exist along with a continuum of functional states between two major subpopulation s of M1 and M2 phenotypes with different functions dependent upon environmental cues (2, 3). M1 Mφs are typically triggered by Th1 cytokines, such as interferon-γ and tumor necrosis factor (TNF)-α, or by bacterial lipopolysaccharide, or by stimulation of granulocyte Mφ colony-stimulating factor (GM-CSF) (2–5). These Mφs produce a large amount of pro-inflammatory cytokines, along with higher expressions of cell surface proteins, such as cluster of differentiation (CD) 54, CD80, CD86, and CD197 (2–5). M1 Mφs elicit strong microbicidal and antitumoral activity, mediate tissue damage, and impair tissue regeneration and wound healing (2–5). In contrast, M2 Mφs are polarized by Th2-associated cytokines, including interleukin (IL)-4, IL-13, and IL-21 in addition to other cytokines, such as IL-10 and Mφ colony-stimulating factor (M-CSF), as well as glucocorticoids, Fc receptors in combination with immunecomplexes, and are often characterized by higher expressions of cell surface scavenger receptors, such as CD163 and CD204, and mannose receptors, including CD206 (2–5). M2 Mφs exhibit the potent phagocytic capacity, scavenge debris, and apoptotic cells, as well as promote tissue repair, wound healing, angiogenesis, and tumor progression (2–7). However, the detailed mechanism remains largely unknown how Mφ induces excessive angiogenesis through the release of pro-angiogenic mediators or the cell–cell interaction with endothelium.

Excessive angiogenesis is a key pathological hallmark of numerous chronic inflammatory diseases such as RA and cancer (8, 9). In the micromilieu of these diseases foci, IL-18, one of a pro-inflammatory cytokine, and osteopontin (OPN), a multifunctional protein linked to various physiological and pathological events, as well as thrombin, the key terminal enzyme of coagulation cascade, are all detected at higher levels (10–15), and have been identified as potent angiogenic mediators (13, 15–20). Note that OPN undergoes proteolytic modification by several proteases like thrombin (21), yielding thrombin-cleaved form of OPN (Thr-OPN), which binds selectively to integrins α4/α9 to exert its functional activity and that these integrins are abundantly expressed in synovial and tumor Mφs (14, 18, 22, 23). Of interest, gene deficiency or blockade of IL-18, OPN, or thrombin ameliorated the progression of RA and cancer, partly due to inhibiting angiogenesis (17–19, 24–26), although it has yet to be determined how these mediators contribute to pathologic angiogenesis.

**Figure 1:**
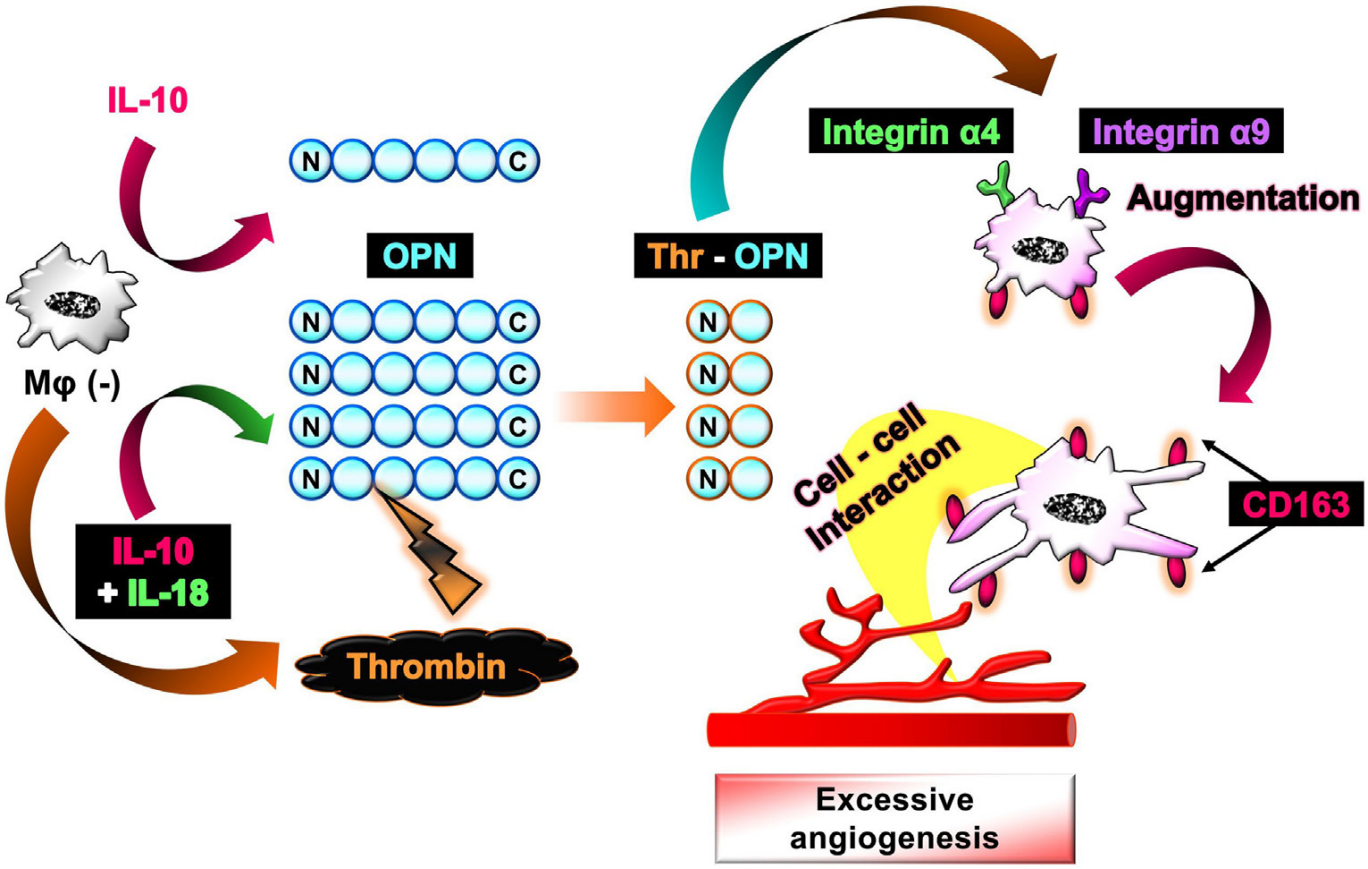
Schematic of the proposed mechanism by which interleukin (IL)-18 amplifies macrophage (Mφ) M2 polarization and its morphological alteration, leading to excessive angiogenesis. IL-18 amplifies IL-10-induced increases in the production of osteopontin (OPN) and thrombin as soluble mediators derived from Mφ, yielding the generation of thrombin-cleaved form of OPN (Thr-OPN). Subsequently, Thr-OPN binds to integrins α4/α9 receptors on Mφ, which in turn augments M2 polarization of Mφ with higher expression of CD163 and its morphological alteration. Furthermore, CD163 may be responsible for mediating the direct cell–cell interaction between these Mφs and endothelial cells, ultimately resulting in the excessive angiogenesis.

The aim of this study is to investigate the mechanism by which Mφ induces excessive angiogenesis in micromilieu containing IL-18 in addition to visualize the temporal behavior and morphological alteration in Mφ during angiogenesis. As a result, we discovered here that IL-18 acts synergistically with IL-10 to amplify the production of OPN and thrombin as soluble mediators derived from Mφ, yielding Thr-OPN generation, which in turn, augments M2 polarization of Mφ with characteristics of increasing CD163 expression in association with morphological alteration. Furthermore, CD163 may be responsible for mediating the direct cell–cell interaction between these Mφs and endothelial cells, ultimately resulting in the excessive angiogenesis (Figure 1).

## Materials and Methods

### Cell Cultures

The mouse leukemic monocyte Mφ cell line, RAW264.7 cells (DS Pharma Biomedical), and the mouse endothelial cell line, b.End5 cells (DS Pharma Biomedical), were cultured in Dulbecco’s modified Eagle’s medium supplemented with 2 mM l-glutamine and 10% heat-inactivated fetal bovine serum. Cells were maintained at 37°C in a humidified atmosphere of 5% CO_2_.

### Flow Cytometric Analysis

RAW264.7 cells were seeded at 5.0 × 10^4^ cells in 24-well plates followed by treatment with recombinant mouse TNF-α (5 ng/mL; R&D systems, 410-MT), IL-10 (10 ng/mL; Sigma-Aldrich, I3019), IL-18 (0.1–200 ng/mL; Medical & Biological Laboratories, B002-5), IL-13 (0.1–200 ng/mL; R&D systems, 413-ML), and IL-33 (0.1–200 ng/mL; R&D systems, 3626-ML) either alone or in combination. In some experiments, hirudin, a thrombin inhibitor (1 µg/mL; Raybiotech, 228-10710), or neutralizing antibodies (Abs) either against OPN (3 µg/mL; R&D systems, AF808), integrin α4 (10 µg/mL; R&D systems, BBA37), integrin α9 (10 µg/mL; R&D systems, AF3827), vascular endothelial growth factor (VEGF) (10 µg/mL; R&D systems, AF493-NA), or M-CSF (5 µg/mL; R&D Systems, MAB4161), or their isotype-matched control Abs were added concomitantly with the above cytokines. After incubating for 24 h at 37°C under 5% CO_2_, cells were harvested and rinsed with FACS wash buffer consisting of phosphate-buffered saline (PBS) supplemented with 2.5% normal horse serum, 0.1% sodium azide, and 10 mM HEPES followed by centrifugation (500 × *g*, 5 min, 4°C). Cells were then stained with anti-mouse Abs against phycoerythrin (PE)-conjugated CD 54 (60 ng; BioLegend, 116108), allophycocyanin (APC)-conjugated CD86 (6.0 ng; Miltenyi Biotec, 130-102-558), fluorescein isothiocyanate (FITC)-conjugated CD68 (25 ng; Bio-Rad Laboratories, MCA1957F), FITC-conjugated CD163 (200 ng; Bioss, bs-2527R-FITC), FITC-conjugated CD206 (200 ng; Bio-Rad Laboratories, MCA2235F), APC-conjugated M-CSF1 receptor (M-CSF1R) (60 ng; BioLegend, 135510), Alexa Fluor 647-conjugated IL-18Rβ (80 ng; Bioss, bs-2616R-A647), FITC-conjugated integrin α4 (130 ng; Thermo Fisher Scientific, 11-0492-82), or PE-conjugated integrin α9 (0.50 µL; Thermo Fisher Scientific, PA5-46896) for 30 min at 4°C. After rinsing with FACS wash buffer followed by centrifugation (500 × *g*, 5 min, 4°C), 300 µL of PBS was added to the residue followed by staining with propidium iodide (PI) (2 µg/mL; Dojindo Laboratories, P378) to exclude PI-positive dead cells. Thereafter, cells were analyzed with a CantoII FACS (BD Biosciences). Data were processed using BD FACSDiva software (BD Biosciences) to determine the mean fluorescence intensity (MFI) of each surface protein.

In the experiment to check the duration of Mφ polarization status after cessation of cytokine stimuli, RAW264.7 cells were seeded at 5.0 × 10^4^ cells in 24-well plates, after which incubated with either TNF-α (5 ng/mL) or IL-10 (10 ng/mL) in the presence or absence of IL-18 (100 ng/mL) for 24 h at 37°C under 5% CO_2_. The cells were then rinsed with PBS to remove cytokines and supplemented with fresh medium, followed by incubation for 0, 24, or 48 h at 37°C under 5% CO_2_. Subsequent procedures were conducted as described above.

### Cell Viability Assay of RAW264.7 Cells after Cytokines Stimuli

RAW264.7 cells were seeded at 5.0 × 10^4^ cells in 24-well plates and stimulated by either TNF-α (5 ng/mL) or IL-10 (10 ng/mL) alone or each combined with different concentrations of IL-18 from 0.1 to 200 ng/mL. After incubating for 24 h at 37°C under 5% CO_2_, cells were harvested and rinsed twice with FACS wash buffer followed by centrifugation (500 × *g*, 5 min, 4°C). Thereafter, the supernatant was removed, followed by addition of PBS. The remaining cells were stained with PI (2 µg/mL) and were analyzed with a CantoII FACS (BD Biosciences). Data were processed using BD FACSDiva software (BD Biosciences) to determine the percentage of PI-positive dead cells in each Mφ subset. Note that the concentrations of IL-18 used in this study are appropriate for *in vitro* experiments as described previously (27–33).

### Dextran Phagocytosis Assay

RAW264.7 cells were seeded at 5.0 × 10^4^ cells in 24-well plates followed by treatment with IL-10 (10 ng/mL) and IL-18 (100 ng/mL) either alone or in combination for 24 h at 37°C under 5% CO_2_. Then, cells were washed with PBS for two times repeat and incubated with dextran-FITC (500 µg/mL; Santa Cruz Biotechnology, sc263323) for 1 h at 37°C under 5% CO_2_. Thereafter, cells were washed with PBS and harvested followed by centrifugation (500 × *g*, 5 min, 4°C), after which 300 µL of PBS was added to the residue and cells were stained with PI (2 µg/mL) to exclude PI-positive dead cells and subsequently analyzed with a CantoII FACS (BD Biosciences). Data were processed using BD FACSDiva software (BD Biosciences) to determine the MFI of FITC-dextran.

### Matrigel Tube Formation Assay

The Matrigel tube formation assay was performed as described previously (34–36), and its simplified schematic overview is shown in Figure S1 in Supplementary Material. Briefly, 96-well plates were filled with 50 µL Matrigel (Corning, 354234) and allowed to solidify at 37°C for 30 min. Then, b.End5 cells (2.0 × 10^4^ cells/100 μL) were gently seeded on top of the gel. Subsequently, polarized Mφs (see Flow Cytometric Analysis) were rinsed with PBS followed by adjustment of the cell number to 0.5 × 10^4^/100 μL and coculturing with b.End5 cells on Matrigel for 16 h at 37°C under 5% CO_2_. In some experiments, CD163, VEGF, or M-CSF functions were inhibited by adding each neutralizing Ab against CD163 (4 µg/mL; Gene Tex, GTX54458), VEGF (10 µg/mL; R&D systems, AF493-NA), or M-CSF (5 µg/mL; R&D Systems, MAB4161), or respective isotype-matched control Abs to the medium. In another experiment to assess the effect of thrombin antagonist on the tube formation induced by VEGF, b.End5 cells were incubated with media containing different doses of recombinant mouse VEGF (1, 5, 10 ng/mL; R&D systems, 493-MV) in the presence or absence of hirudin (1 µg/mL) or anti-VEGF Ab (10 µg/mL; R&D systems, AF493-NA) on Matrigel in the same condition described above. After incubation, tube-like structures were visualized with 8 µg/mL of calcein acetoxymethylester (AM) (Corning, 354217) and photomicrographs were taken at 5–8 µm intervals for *z*-axis obtained at magnification 100× with a confocal laser C2 microscope (Nikon). The areas and total lengths of the tubes were calculated with NIS-Elements Ar software (Nikon) and evaluated as the degree of tube formation according to previous reports (34–36). Note that b.End5 cells were selected for use in this assay from several endothelial cell lines because they are of mouse origin, the same as RAW264.7 cell. In addition, b.End5 cells can be cultivated continuously in the same medium used for RAW264.7 cell. Although b.End5 cells are derived from brain endothelial cells, this cell line is widely used for the Matrigel tube formation assay to evaluate angiogenesis in brain as well as other regions (37–39) as in the current study.

To examine cell number-dependent changes in the degree of tube formation, b.End5 cells were seeded at 1.0–4.0 × 10^4^ cells/100 μL gently on top of Matrigel followed by incubation for 16 h at 37°C under 5% CO_2_. To assess the effect of cytokine stimuli on the degree of tube formation, b.End5 cells (2.0 × 10^4^ cells/100 μL) were incubated on Matrigel for 16 h at 37°C under 5% CO_2_ in the presence of either TNF-α (5 ng/mL) or IL-10 (10 ng/mL) alone or each combined with IL-18 (0.1–200 ng/mL) instead of Mφs.

To further determine the influence of soluble mediators derived from each Mφ subset on the angiogenesis, we constructed an indirect contact coculture model with transwell plates in the Matrigel tube formation assay. Twenty four-well transwell plates were filled with 200 µL Matrigel and allowed to solidify at 37°C for 30 min. Then, a total of 8.0 × 10^4^ cells/well of b.End5 cells was gently seeded on top of the gel in the lower chamber. Thereafter, polarized RAW264.7 cells (see Flow Cytometric Analysis) were rinsed with PBS, 2.0 × 10^4^ cells/well of which was seeded on the upper chamber inserts followed by the indirect coculture of these cells for 16 h at 37°C under 5% CO_2_. After incubation, tube-like structures were visualized with 8 µg/mL of calcein AM and photomicrographs were taken at 5–8 µm intervals for *z*-axis obtained at magnification 100× with a confocal laser C2 microscope (Nikon). The areas and total lengths of the tubes were calculated with NIS-Elements Ar software (Nikon) and evaluated as the degree of tube formation.

### PKH Labeling

To distinguish between endothelial cells and Mφs in the Matrigel tube formation assay, b.End5 cells were fluorescently labeled with PKH67 (green; Sigma-Aldrich, MINI67) and RAW264.7 cells with PKH26 (red; Sigma-Aldrich, MINI26) according to the manufacturer’s protocol. After that, PKH26-labeled RAW264.7 cells seeded at 5.0 × 10^4^ cells in 24-well plates were treated with IL-10 (10 ng/mL) in combination with IL-18 (100 ng/mL) for 24 h at 37°C under 5% CO_2_. The subsequent procedure was performed in a manner similar to the Matrigel Tube Formation Assay except for the cell numbers of PKH67-labeled b.End5 cells (4.0 × 10^4^/100 μL) and PKH26-labeled Mφ (IL-10 + IL-18) (1.0 × 10^4^/100 μL) when cocultured on Matrigel.

### Cell Viability Assay of b.End5 Cells after Coculturing with Mφ Subsets

PKH67-labeled b.End5 cells were seeded at 4.0 × 10^4^ cells and allowed to adhere to 24-well plates for 4 h at 37°C under 5% CO_2_. Then, 1.0 × 10^4^ cells of Mφ (–), Mφ (IL-10), Mφ (IL-18), or Mφ (IL-10 + IL-18) were overlaid onto fluorescent b.End5 cells and cocultured for 16 h at 37°C under 5% CO_2_. Subsequently, photomicrographs were taken at 2–5 µm intervals for *z*-axis obtained at magnification 100× with a confocal laser C2 microscope (Nikon). The area of PKH67-positive region was calculated with NIS-Elements Ar software (Nikon) and evaluated as the number of surviving b.End5 cells.

### Time-Lapse Live-Cell Imaging of Matrigel Tube Formation Assay

35-mm dish was filled with 800 µL of Matrigel and allowed to solidify at 37°C for 30 min. Subsequently, PKH67 (green)-labeled b.End5 cells (6.0 × 10^5^ cells) were gently seeded on top of the gel, followed by cocultured with PKH26 (red)-labeled Mφ (–), Mφ (IL-10), Mφ (IL-18), or Mφ (IL-10 + IL-18) (1.5 × 10^5^ cells) on Matrigel. After that, 35-mm dish was placed on the stage of an All-in-One fluorescence microscope BZ-X710 (Keyence) equipped with an environmental chamber, which provided 37°C, humidity and 5% CO_2_ conditions. Time-lapse live-cell imaging of Matrigel tube formation assay was obtained at 1–3 µm intervals for the *z*-axis every 10 min over 16 h with a set of green and red emissions at original magnification 200×. Obtained images were processed and analyzed with BZ-X710 software (Keyence).

### Scanning Electron Microscopy (SEM)

RAW264.7 cells seeded at 5 × 10^4^ cells in 24-well plates were incubated in the medium containing IL-10 (10 ng/mL) and IL-18 (100 ng/mL) alone or their combination for 24 h at 37°C under 5% CO_2_ condition. Thereafter, 35-mm dish was filled with 800 µL of Matrigel and allowed to solidify at 37°C for 30 min. Then, b.End5 cells (6.0 × 10^5^ cells) were gently seeded on top of the gel. Subsequently, Mφ (–), Mφ (IL-10), Mφ (IL-18), or Mφ (IL-10 + IL-18) were rinsed with PBS followed by coculture of b.End5 cells with each subset of Mφs (1.5 × 10^5^ cells) on Matrigel for 4 h at 37°C under 5% CO_2_ condition. For SEM analysis, 35-mm dishes were rinsed with 0.1 M PBS pH 7.4 at 4°C for 60 min and were fixed with 2.5% (v/v) glutaraldehyde in 0.1 M PBS pH 7.4 at 4°C for overnight. After rinsing with 0.1 M PBS pH 7.4 at 4°C for 60 min, cells were post-fixed with 1% (w/v) osmium tetroxide or at 4°C for 2 h followed by washing with 0.1 M PBS pH 7.4 at 4°C for 60 min, dehydration in an ascending ethanol series (50, 70, 80, 80, 90, and 95% for 15 min each and three times with 100% ethanol for 15 min each). The samples were coated with platinum-palladium freeze-dried three times with *t*-butyl alcohol for 20 min each and observed using a SEM SU3500 (Hitachi High-Technologies) at 15 kV.

### RNA Extraction and the Real-time Reverse Transcription Polymerase Chain Reaction (RT-PCR)

After treatment of RAW264.7 cells (2.0 × 10^5^ cells in six-well plates) with either IL-10 (10 ng/mL) or IL-18 (100 ng/mL) alone or their combination for 24 h at 37°C under 5% CO_2_, total RNA was extracted using Trizol (Thermo Fisher Scientific). The quality and quantity of total RNA were measured with a NanoDrop One spectrophotometer (Thermo Fisher Scientific) and 100–1,000 ng of total RNA was used as a template. Real-time quantitative RT-PCR amplifications were performed in 96-well optical reaction plates using an Applied Biosystems 7900HT Fast Real Time PCR System (Thermo Fisher Scientific) with a One Step SYBR Prime Script PLUS RT-PCR kit (Takara Bio, RR096A) and the gene-specific primers (all purchased from Takara Bio) at 42°C for 5 min, thereafter 95°C for 10 s followed by 40 cycles of 95°C for 5 s and 60°C for 30 s, finally 95°C for 15 s. The amounts of target mRNA, *Spp1* (the gene coding OPN), *Prothrombin, Mmps-2, -3, -7*, and *-9* were normalized to the level of glyceraldehyde-3-phosphate dehydrogenase (*Gapdh*) mRNA amplified from the same sample, and then to untreated control mRNA. Data were analyzed with the calibration curve method using 7900HT Fast system SDS software version 2.4 (Thermo Fisher Scientific). The gene-specific primer sequences are given in Table 1.

**Table 1:** Gene specific primers sequences.

Gene	Primer sequence
*Gapdh* (forward)	TGTGTCCGTCGTGGATCTGA
*Gapdh* (reverse)	TTGCTGTTGAAGTCGCAGGAG
*Spp1* (forward)	TACGACCATGAGATTGGCAGTGA
*Spp1* (reverse)	TATAGGATCTGGGTGCAGGCTGTAA
*Prothrombin* (forward)	CCTGGTGCTACACCACAGATCCTA
*Prothrombin* (reverse)	GTGACAGATTGTCCTTGGAACCTC
*Mmp-2* (forward)	AGAACTTCCGATTATCCCATGATGA
*Mmp-2* (reverse)	TGACAGGTCCCAGTGTTGGTG
*Mmp-3* (forward)	CTGGACCAGGGATTAATGGAGA
*Mmp-3* (reverse)	TCATGAGCAGCAACCAGGAA
*Mmp-7* (forward)	GGCGGAGATGCTCACTTTGAC
*Mmp-7* (reverse)	AATTCATGGGTGGCAGCAAAC
*Mmp-9* (forward)	GCCCTGGAACTCACACGACA
*Mmp-9* (reverse)	TTGGAAACTCACACGCCAGAAG

### Protein Isolation and Western Blotting Analysis

RAW264.7 cells seeded at 2.0 × 10^5^ cells in six-well plates were treated either alone or in combination with IL-10 (1–100 ng/mL) and IL-18 (1–100 ng/mL), or concomitantly with hirudin (1 µg/mL) for 24 h at 37°C under 5% CO_2_. Then, cells were harvested and washed with PBS, and subsequently lysed in radio-immunoprecipitation assay (RIPA) buffer containing protease inhibitors for 30 min on ice. The supernatant of the resulting suspension was obtained after centrifugation (16,000 × *g*, 30 min, 4°C) and collected as the total cell lysate. The total protein concentration was quantified using a Bradford protein assay kit (Bio-Rad Laboratories).

Total lysates of RAW264.7 cells were diluted with an equal volume of 2× Laemmli buffer containing 0.5 M Tris–HCl (pH 6.8), 10% sodium dodecyl sulfate (SDS), 12% β-mercaptoethanol, 20% glycerol, and 0.1% bromophenol blue, then heated for 5 min at 97°C. 10 µg of total protein were loaded and separated by SDS-polyacrylamide gel electrophoresis followed by transfer onto a nitrocellulose membrane (Bio-Rad Laboratories). The membrane was incubated in blocking buffer containing 5% non-fat dry milk (Wako Pure Chemical) or 10% bovine serum albumin (used only with OPN N-Half; Wako Pure Chemical) in PBS-T (PBS pH 7.6 with 0.1% Tween-20) for 60 min at room temperature. The membrane was then probed with primary anti-mouse Abs against M-CSF (5 µg/mL; R&D Systems, MAB4161), OPN (0.05 µg/mL; R&D Systems, AF808), OPN N-Half [34E3 which reacts specifically with the N-terminal fragment of OPN cleaved by thrombin (0.05 µg/mL; Immuno-Biological Laboratories, 11108)], thrombin (1:1,000 dilution; Abcam, ab92621), VEGF (1:4,000 dilution; Abcam, ab46154), VEGF receptor (VEGFR) 1 (1:1,000 dilution; Abcam, ab32152), and VEGFR2 (1:1,000 dilution; Cell Signaling Technology Japan, 2479) as well as actin (1:2,000 dilution; Santa Cruz Biotechnology, sc-1616) or GAPDH (1:20,000 dilution; Merck Millipore, MAB374) used as internal controls in respective blocking buffer at 4°C overnight. Blots were then washed with PBS-T and incubated with horseradish peroxidase-conjugated secondary Abs against goat IgG (1:2,000 dilution for OPN and actin; 14-13-06), mouse IgG (1:4,000 dilution for OPN N-Half and GAPDH; 074-1806), rabbit IgG (1:4,000 dilution for thrombin, VEGF, VEGFR1, and VEGFR2; 074-1506), or rat IgG (1:1,000 dilution for M-CSF; 5220-0284) (all bought from Kirkegaad and Perry Laboratories) in respective blocking buffer for 60 min at room temperature. After washing with PBS-T, the immune-complexes were visualized using Pierce Western Blotting Substrate (Thermo Fisher Scientific, 32106). The signal intensity of the immune reactive bands was detected with Amersham Imager 600 (GE Health Care Japan). The relative band intensities were quantified using Image Quant TL software (GE Health Care Japan). Values for M-CSF, OPN, OPN N-Half, thrombin, and VEGF relative to GAPDH, or values for VEGFR1 and VEGFR2 relative to actin were normalized to untreated cells.

### Comprehensive Protein Analysis

RAW264.7 cells seeded at 2.0 × 10^5^ cells in six-well plates were stimulated with IL-10 (10 ng/mL) in the presence or absence of IL-18 (100 ng/mL) for 24 h at 37°C under 5% CO_2_. Thereafter, cells were harvested and placed in a clean test tube, and centrifuged (500 × *g*, 5 min, 4°C). The supernatant was then removed, and the cells washed with PBS followed by centrifugation (500 × *g*, 5 min, 4°C). After removing the supernatant, the cells were washed with PBS and further centrifuged (3,400 × *g*, 5 min, 4°C) followed by elimination of the supernatant. The remaining cells were lysed in RIPA buffer containing protease inhibitors for 30 min on ice. Subsequently, the cell suspension was centrifuged (16,000 × *g*, 30 min, 4°C), and the supernatant was transferred into a clean test tube as total cell lysate. The total protein concentration of each sample was quantified by the Bradford method (40) and adjusted to 200 µg protein/sample. A comprehensive protein analysis using the Proteome Profiler Mouse XL Cytokine Array kit (R&D Systems, ARY028) was conducted according to the manufacturer’s protocol.

### Confocal Laser Scanning Immunofluorescence Analysis

After adhering RAW264.7 cells seeded at 1 × 10^5^ cell/dish to 35-mm glass bottom dish (Matsunami Glass) for 2 h, cells were incubated with IL-10 (10 ng/mL) in the presence or absence of IL-18 (100 ng/mL) for 24 h at 37°C under 5% CO_2_. Thereafter, cells were washed with PBS and fixed with 4% formaldehyde (PFA) at room temperature for 30 min followed by washing with PBS-T. Subsequently, cells were incubated in blocking buffer containing 1% BSA, 0.3 M glycine in PBS-T at room temperature for 1 h to permeabilize cell membrane and to block non-specific protein–protein interactions. After that, they were incubated with primary Abs directed against OPN (1:50 dilution; R&D Systems, AF808), thrombin (1:50 dilution; Abcam, ab92621), integrin α4 (1:50 dilution; R&D Systems, BBA37), integrin α9 (1:50 dilution; R&D Systems, FAB3827), or CD163 (1:20 dilution; Bioss, bs-2527R) in blocking buffer at 4°C overnight. After rinses in PBS-T, cells were incubated with Alexa Fluor 488-conjugated secondary Ab against rabbit IgG (1:1,000 dilution for thrombin and CD163; Thermo Fisher Scientific, A-11008), Alexa Fluor 488-conjugated secondary Ab against mouse IgG (1:500 dilution for integrin α4; Thermo Fisher Scientific, A-11001), or Alexa Fluor 594-conjugated secondary Ab against goat IgG (1:1,000 dilution for OPN, 1:500 dilution for integrin α9; Thermo Fisher Scientific, A-11058) at room temperature for 1 h. Then, cells were washed with PBS-T followed by counterstaining with 4′,6-diamidine-2′-phenylindole dihydrochloride (DAPI) (0.72 µM; Sigma-Aldrich, D9542), thereafter photomicrographs were taken at 0.6–0.8 µm intervals for *z*-axis at original magnification 400× with a confocal laser C2 microscope (Nikon). The two- or three-dimensional images were reconstructed from obtained pictures using NIS-Elements Ar software (Nikon).

### Statistical Analysis

Data are presented as means ± SEM. Statistical analysis was performed using Prizm version 3 software (GraphPad Software). Statistical significance was assessed using a one-way analysis of variance followed by Dunnett’s or Tukey’s test for multiple comparisons. A value of *P* < 0.05 was considered as significant.

## Results

### IL-18 Amplifies Mφ M2 Polarization and Angiogenic Capacity

According to the recent proposal of nomenclature for Mφ subsets linked to the stimulation scenarios (41), Mφs treated with medium (untreated), TNF-α, IL-10, IL-18, TNF-α + IL-18, or IL-10 + IL-18 were henceforth described as Mφ (–), Mφ (TNF-α), Mφ (IL-10), Mφ (IL-18), Mφ (TNF-α + IL-18) or Mφ (IL-10 + IL-18), respectively.

**Figure 2:**
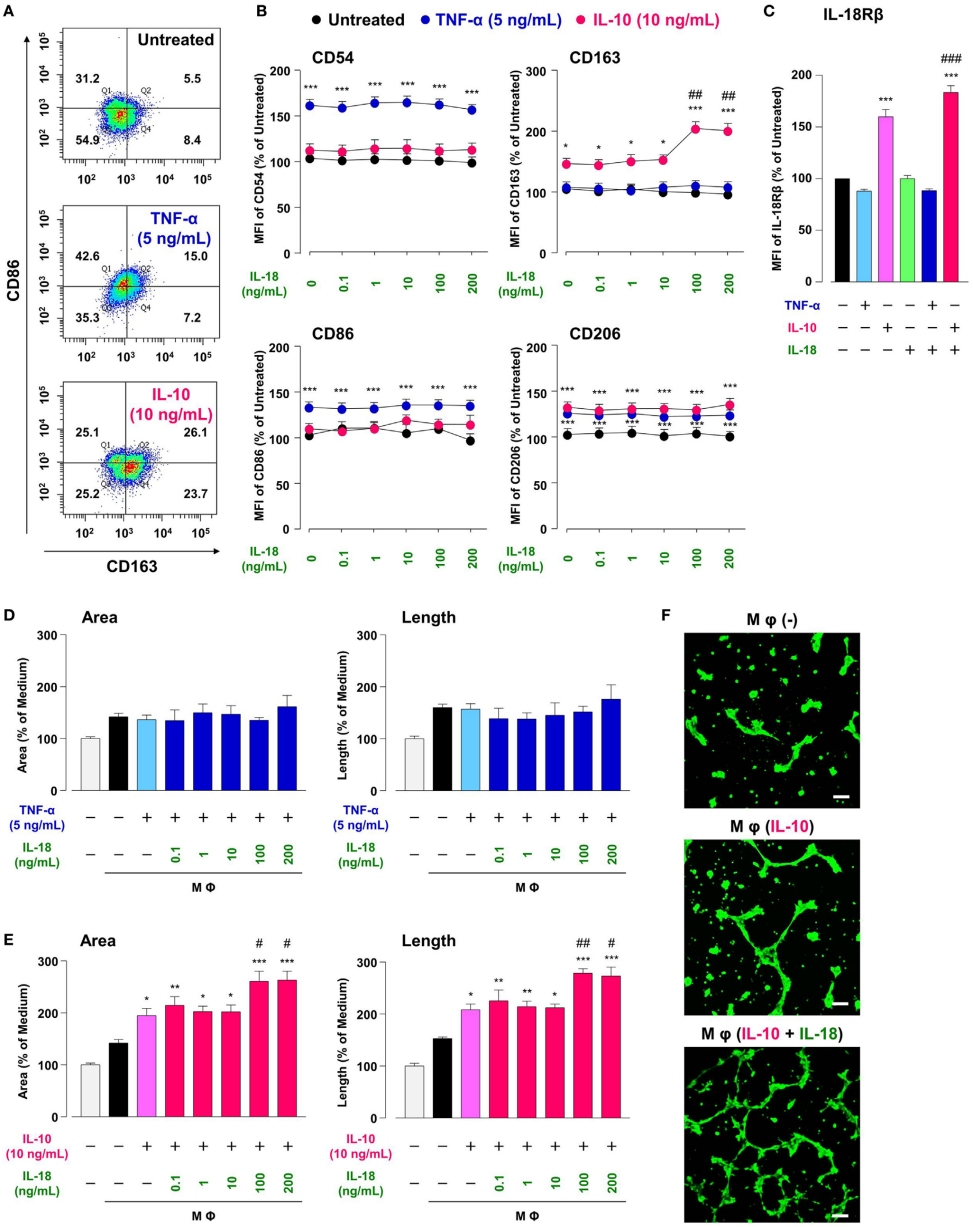
Interleukin (IL)-18 amplifies macrophage (Mφ) M2 polarization and angiogenic capacity. **(A)** Representative FACS density plots for the expression of CD86 and CD163 in each Mφ subset. Upper, Mφ (–); middle; Mφ [tumor necrosis factor (TNF)-α]; lower, Mφ (IL-10). The numbers in each quartile of the plots are percentages of each cell population. **(B)** Relative mean fluorescence intensities (MFIs) of CD54, CD86, CD163, and CD206 in each Mφ subset were measured by FACS analysis, *n* = 3 (***p < 0.001, *p < 0.05 vs. untreated, ^##^*p* < 0.01 vs. IL-10 alone). **(C)** Relative MFI of IL-18Rβ in each Mφ subset was determined by FACS analysis, *n* = 4 (***p < 0.001 vs. untreated, ^###^ *p* < 0.001 vs. IL-10 alone). **(D,E)** The total areas and lengths of tube-like structures measured by the Matrigel tube formation assay where b.End5 was cocultured with each Mφ subset, *n* = 6 (***p < 0.001, **p < 0.01, *p < 0.05 vs. untreated, ^##^*p* < 0.01, ^#^*p* < 0.05 vs. IL-10 alone). **(F)** Representative pictures of tube-like structures visualized by calcein acetoxymethylester staining. Scale bar represents 100 µm. All data are presented as means ± SEM and were analyzed by a one-way ANOVA followed by Tukey’s test.

Treatment of RAW264.7, the mouse Mφ-like cell line, with TNF-α or IL-10 increased Mφ M1 or M2 markers expression, respectively, in comparison with Mφ (–), resulting in a shift of population in CD86-positive/CD163-negative region by TNF-α or opposite shift by IL-10 (Figures 2A,B; Figures S2A,B in Supplementary Material). A moderate increase in CD206 level by TNF-α is partly supported by a previous study showing the ability of TNF-α to slightly induce Mφ M2 marker (42). These results are in accordance with the widely accepted concept that M1 or M2 phenotypes are induced by pro- or anti-inflammatory cytokines, respectively (3, 4). Thus, certain cytokines milieu may skew Mφs toward both M1/M2 phenotypes. Additionally, the concentrations of cytokines that selectively polarized Mφs toward M1/M2-like phenotypes (Figures S2A,B in Supplementary Material) were similar to previous reports (34, 35). Of note, IL-10 but not TNF-α increased the surface CD68 expressions in a concentration-dependent manner (Figures S2A,B in Supplementary Material), which might partly explain the fact that the number of CD68-positve Mφs was markedly increased in the foci of chronic inflammatory diseases like RA (43–46)and a variety of cancer (47–49).

Interleukin-18 alone influenced none of M1/M2 marker expression at concentrations from 0.1 to 200 ng/mL (Figure 2B), high concentrations of which are used in recent papers (16, 27). While IL-18 in the same concentration range showed no impact on alteration in M1/M2 markers induced by TNF-α (Figure 2B), IL-18 at concentrations of 100 and 200 ng/mL but not at others significantly enhanced an increase in CD163 level (50% effective dose: 60.4 ng/mL) by IL-10, without any influence on other markers (Figure 2B). These differences might arise from the present result that IL-10-induced an increase in IL-18 receptor level was significantly potentiated by concomitant use of IL-18, while IL-18 alone or in combination with TNF-α had no influence (Figure 2C). In fact, IL-18 exerts opposite functions on Mφ M1 polarization process dependent upon surrounding environmental cues (27, 50). Additionally, the concomitant use of IL-18 had no effect on decrease in CD68 level induced by IL-10 (Figure S2C in Supplementary Material). Taken together, our findings provide a new aspect of IL-18 to amplify Mφ M2 polarization in addition to an important role of CD163 in the action of IL-10 and IL-18 (Figure 1). Note that cytokines combination used here had no influence on the viability of RAW264.7 (Figure S3 in Supplementary Material) and that significant changes in M1/M2 marker expressions (Figures 2A,B) were persisted up to 24 h after cessation of each cytokine alone or in combination, but were declined to the control level at 48 h (Figure S4 in Supplementary Material).

Similar to the approach of previous studies (34, 35), we conducted the Matrigel tube formation assay using b.End5, the mouse endothelial cell line. Note that the simplified experimental overview was described in Figure S1 in Supplementary Material and that the degree of tube formation was dependent upon the number of b.End5 cells seeded on Matrigel (Figures S5A,B in Supplementary Material). Coculture of b.End5 cells with Mφ (–) increased the area and length of tube-like structures moderately compared with the control group without RAW264.7. There was no difference in the degree of tube formation between Mφ (–), Mφ (TNF-α), and Mφ (TNF-α + IL-18) (Figure 2D). By contrast, tube formation was increased drastically by culturing with Mφ (IL-10) compared to Mφ (–), which is consistent with the general concept that M2-like Mφs strongly induce angiogenesis (35, 51). Moreover, IL-10 when combined with IL-18 at 100 or 200 ng/mL, but not with other concentrations, significantly augmented the tube formation compared to Mφ (IL-10) (Figures 2E,F); therefore, we used IL-18 at 100 ng/mL in the subsequent experiments. Together, IL-18 synergized with IL-10 to augment the angiogenic capacity of Mφs, which might represent the mechanism underlying excessive angiogenesis elicited by IL-18 (Figure 1). Note that in the absence of RAW264.7, direct stimulation of b.End5 cells with TNF-α or IL-10 alone or in combination with IL-18 at any concentration used above, showed little impact on tubulogenesis (Figures S5C,D in Supplementary Material). Of particular note, indirect cocultures of b.End5 cells with Mφ (–), Mφ (IL-10), Mφ (IL-18), or Mφ (IL-10 + IL-18) never facilitated the formation of tube-like structures, implying that secretory mediators derived from each Mφ subset had an insufficient effect to enhance the angiogenesis in this experiment (Figure S5E in Supplementary Material). Another importance is that the phagocytic activity of Mφ (IL-10) was higher than that of Mφ (–) (Figure S6A in Supplementary Material), which is in line with previous finding (52). By contrast, treatment with IL-18 exerted little influence on the phagocytosis of Mφ (–) and Mφ (IL-10) (Figure S6A in Supplementary Material). Moreover, none of Mφ subsets used in this analysis exhibited cytotoxic activity against b.End5 cells after coculture of these cells (Figure S6B in Supplementary Material).

### Characteristic Behavior of Mφs during Angiogenesis

**Figure 3:**
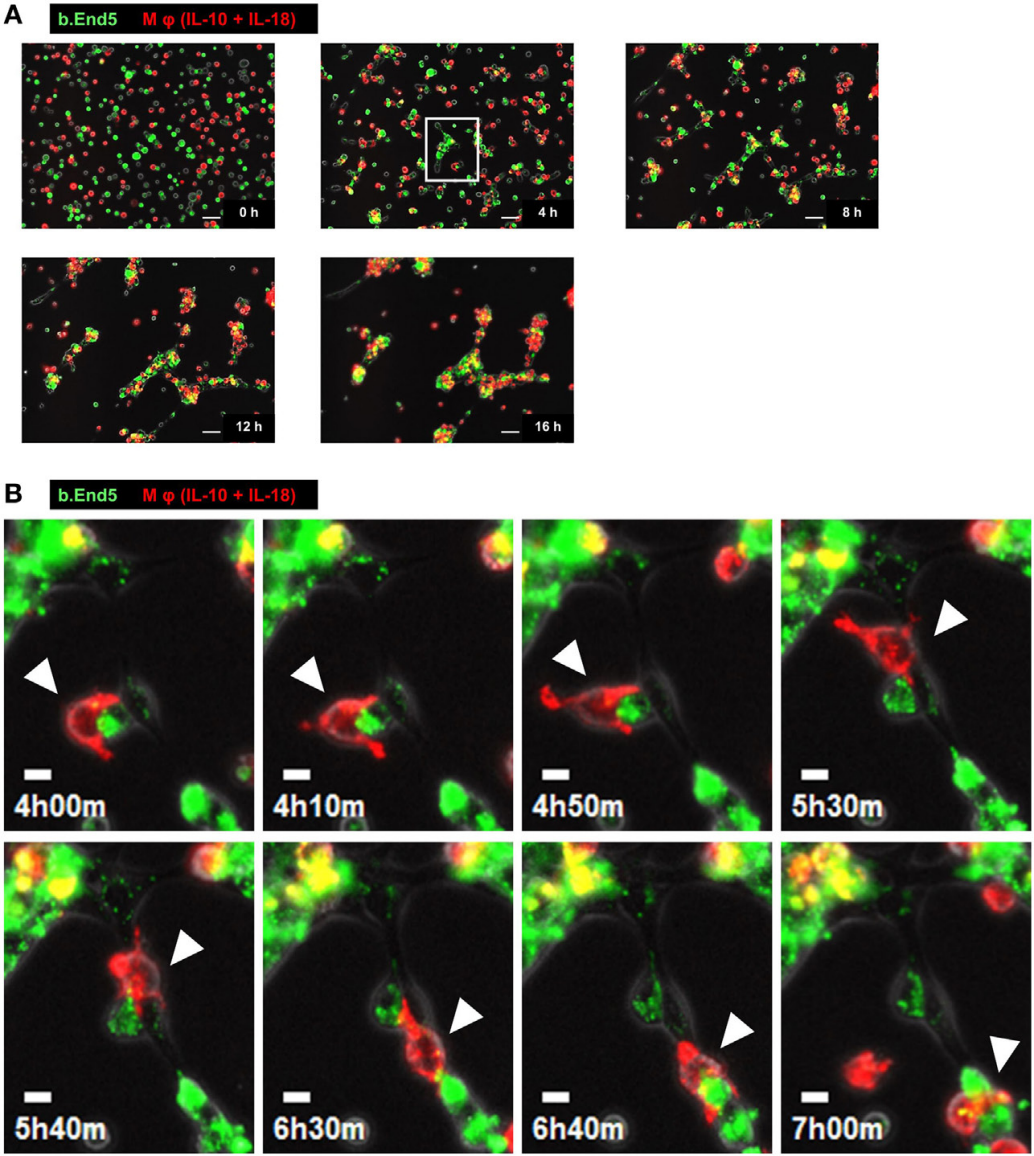
Characteristic behavior of macrophages (Mφs) during angiogenesis. **(A)** Representative series of time-lapse images at 4 h intervals from 0 to 16 h extracted from Video S1 which shows live-cell imaging of Matrigel tube formation assay where endothelial cells (green) and Mφs [interleukin (IL)-10 + IL-18] (red) were cocultured. Scale bar represents 50 µm. **(B)** Higher magnification images of white rectangle region in panel **(A)** were reconstructed from 4 h 00 min to 7 h 00 min in Video S5 in Supplementary Material. The time elapsed after starting the movie is indicated in hours:minutes in the bottom left of each panel. White arrowheads highlight the characteristic behavior of Mφ (IL-10 + IL-18) as well as the cell–cell interaction with endothelium in respective image. Scale bar represents 10 µm.

To understand the temporal behavior of Mφs during angiogenesis, we performed time-lapse live-cell imaging experiments with the Matrigel tube formation assay. After starting the observation from 0 to 3 h, PKH67 (green)-labeled b.End5 cell was in contact with each other from far and wide. Subsequently, vascular segments consists of b.End5 cells connected to each other through the help of PKH26 (red)-labeled Mφs (IL-10 + IL-18) and Mφs (IL-10) mediated *via* direct cell–cell interaction from 3 to 8 h, driving a rapid induction of tubulogenesis, whereas Mφs (–) and Mφs (IL-18) hardly move or communicate with endothelial cells. Thereafter, numerous Mφs (IL-10 + IL-18) apparently gathered around the leading edge of the growing vascular network and/or branching points of vasculature where they interacted with endothelium, allowing vascular tube to get thicker and thicker. The acceleration of tubulogenesis was almost completed until 12 h and reached a plateau phase toward the end of observation period (Figure 3A; Figure S7 and Videos S1–S4 in Supplementary Material). However, it remains unsolved whether each subset of Mφs accumulated at the sites of vessel fusion or junction in which they embraced vascular tubes merely form cell aggregates or have any functional properties. Intriguingly, series of high magnification images extracted from Video S1 revealed that a part of Mφs (IL-10 + IL-18) spread pseudopodia wide apart, hereby captured and brought endothelium into close apposition of vascular tubes probably through the direct cell–cell contact. Subsequently, Mφs (IL-10 + IL-18) attained supportive role to keep endothelium at capillaries by bridging between endothelial cells, leading to angiogenic event such as vascular sprouting and/or junction. This Mφ subset remained in contact with vessels for at least some time after vascular tubes had fused to form the intersection albeit moving to another parts of tube network (Figure 3B; Video S5 in Supplementary Material).

### Ultrastructural Analysis of Cell–Cell Interaction between Mφs (IL-10 + IL-18) and Endothelia

**Figure 4:**
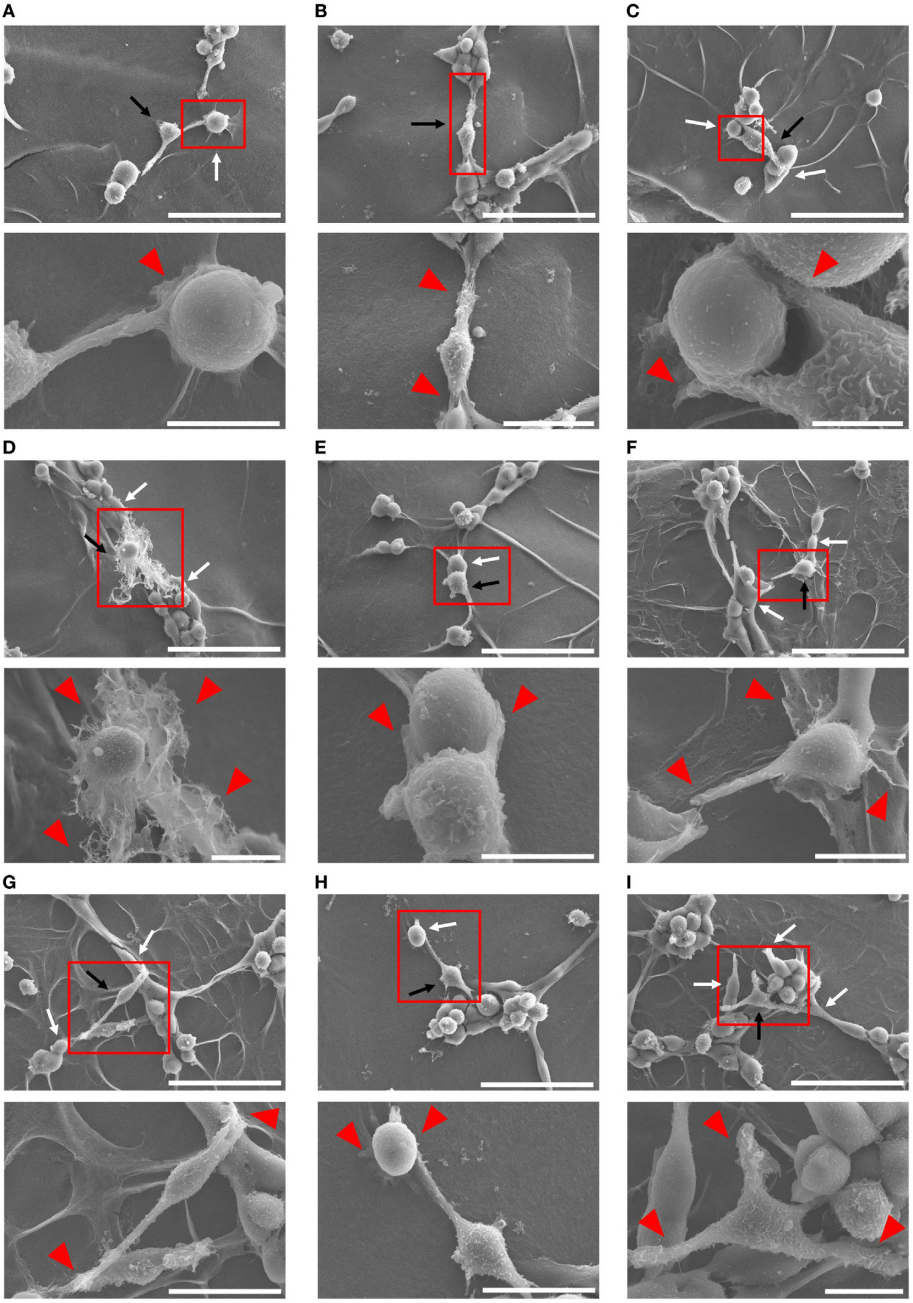
Ultrastructural analysis of cell–cell interaction between macrophages (Mφs) [interleukin (IL)-10 + IL-18] and endothelia. SEM images were obtained at 4 h after coculture of b.End5 with Mφs (IL-10 + IL-18) on Matrigel. Lower images are magnified regions from red rectangles in the corresponding upper panels. Magnification and scale bars: **(A)** upper, ×1.0 K, 50 µm; Lower, ×5.0 K, 10 µm; **(B)** upper, ×1.0 K, 50 µm; lower, ×2.0 K, 20 µm; **(C)** upper, ×1.0 K, 50 µm; lower, ×8.0 K, 5 µm; **(D)** upper, ×1.0 K, 50 µm; lower, ×3.0 K, 10 µm; **(E)** upper, ×1.0 K, 50 µm; lower, ×5.0 K, 10 µm; **(F)** upper, ×1.0 K, 50 µm; lower, ×4.0 K, 10 µm; **(G)** upper, ×1.0 K, 50 µm; lower, ×2.5 K, 20 µm; **(H)** upper, ×1.0 K, 50 µm; lower, ×2.5 K, 20 µm; **(I)** upper, ×1.0 K, 50 µm; lower, ×3.5 K, 10 µm; respectively. Black or white arrows indicate Mφs or endothelial cells, respectively. Red arrowheads indicate pseudopodia of Mφs interacting with endothelial cells.

Because Mφs have been demonstrated to associate tightly with capillaries in the progression of angiogenesis (53, 54), we tried to further confirm the cell–cell interaction between each Mφ subset and endothelium by means of SEM analysis. After 4 h coculture, Mφs (IL-10 + IL-18) appeared to twist endothelium using their pseudopodia near the site at which vessel sprouting and/or fusion occur (Figure 4A) and to bridge the vascular gap with bidirectionally spreading pseudopodia for connecting the neighboring vessel segments (Figures 4B–D). Moreover, in vascular network these Mφs were frequently found at the tip of tube-like structure where they embraced and/or brought endothelium with pseudopodia into the leading edge of growing vessels (Figures 4E–G) as well as at branching points where they attracted endothelium with pseudopodia from adjacent region to vessel junction (Figures 4H,I); therefore, they seemed likely to destine the direction for outgrowth. Note that the morphological alterations of Mφs (IL-10 + IL-18) observed in Figures 4A–I appear to reflect the results of Figure 3B. While other subsets of Mφs, including Mφs (–) (Figures S8A–D in Supplementary Material), Mφs (IL-10) (Figures S8E–H in Supplementary Material), and Mφs (IL-18) (Figures S8I–L in Supplementary Material) also embraced endothelium, these Mφ subsets were found at the tip of tube-like structure and at the branching points less often than Mφ (IL-10 + IL-18). Of note, they rarely twisted endothelium and brought the vascular gap, despite the existence of spreading pseudopodia.

### OPN Drives Enhancement in Mφ M2 Polarization and Angiogenic Capacity

**Figure 5:**
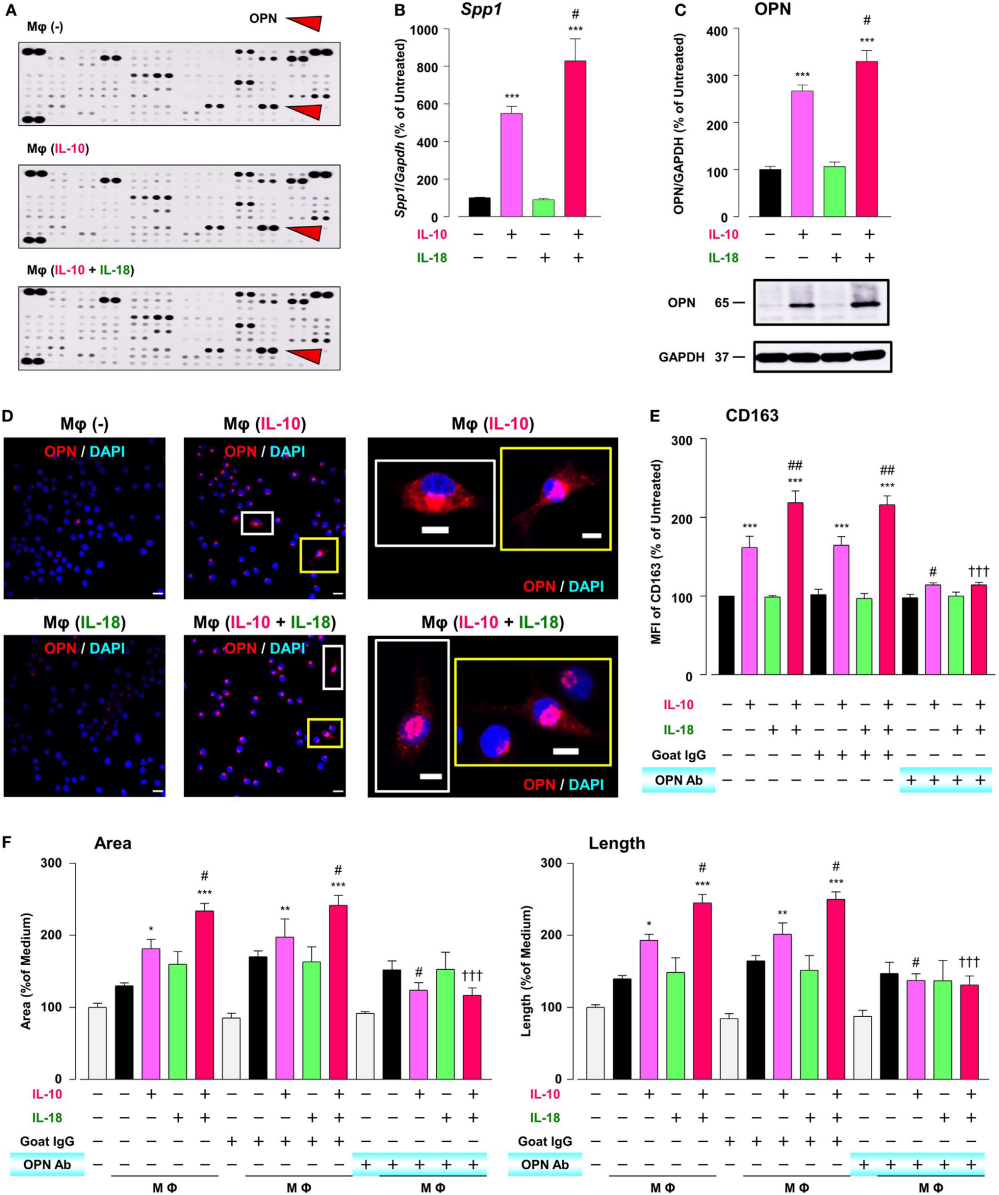
Osteopontin (OPN) drives enhancement in macrophage (Mφ) M2 polarization and angiogenic capacity. **(A)** Representative images of protein expression profiles obtained by comprehensive protein array in each Mφ subset. Red arrowheads indicate OPN. **(B)** The mRNA expression level of *Spp1* relative to glyceraldehyde-3-phosphate dehydrogenase (*Gapdh*) was analyzed by real-time reverse transcription polymerase chain reaction in each Mφ subset and was normalized to Mφ (–), *n* = 6 [***p < 0.001 vs. untreated, ^#^*p* < 0.05 vs. interleukin (IL)-10 alone]. **(C)** The protein expression level of OPN relative to GAPDH was measured by western blotting and was normalized to Mφ (–), *n* = 10. Lower panels are typical images of each protein (***p < 0.001 vs. untreated, ^#^*p* < 0.05 vs. IL-10 alone). **(D)** Representative confocal laser scanning immunofluorescence overlay images of OPN (red) and DAPI (blue) in each Mφ subset. Scale bar represents 20 µm. Images in the right row are magnified regions from white or yellow rectangles in the panels of corresponding groups. Scale bar represents 10 µm. **(E)** Relative mean fluorescence intensity (MFI) of CD163 was measured by FACS analysis in each Mφ subset. An anti-OPN antibody (Ab) and its isotype-matched control Ab were used at 3 µg/mL, *n* = 4 (***p < 0.001 vs. untreated, ^##^*p* < 0.01, ^#^*p* < 0.05 vs. IL-10 alone, ^†††^*p* < 0.001 vs. IL-10 + IL-18). **(F)** The total areas and lengths of tube-like structures were determined by the Matrigel tube formation assay where b.End5 was cocultured with each Mφ subset, *n* = 12 (***p < 0.001, **p < 0.01, *p < 0.05 vs. untreated, ^#^*p* < 0.05 vs. IL-10 alone, ^†††^*p* < 0.001 vs. IL-10 + IL-18). All data are expressed as means ± SEM and were analyzed by a one-way ANOVA followed by Tukey’s test.

To explore the mechanism by which IL-18 augments IL-10-induced Mφ M2 polarization and angiogenic potency, we focused on OPN, a pleiotropic cytokine produced by various cells including Mφ (11), according to the results of comprehensive protein analysis showing that OPN levels were obviously increased in Mφ (IL-10), which was further enhanced in Mφ (IL-10 + IL-18) (Figure 5A). Because OPN expression is markedly elevated in the micromilieu of several chronic inflammatory conditions such as RA and cancer (14, 55) and genetic deficiency or blockade of OPN ameliorates the development of these diseases (17, 18), OPN may be of interest as a fruitful target for various diseases. The expression level of *Spp1*, the gene coding OPN (Figure 5B) and that of OPN protein (Figure 5C; Figure S9A in Supplementary Material) as well as intracellular staining intensity of OPN (red) (Figure 5D) were dramatically increased in Mφ (IL-10) when compared to Mφ (–), which were further potentiated in Mφ (IL-10 + IL-18) (Figures 5B–D). It is noteworthy that the concurrent use of IL-18 (1–100 ng/mL) potentiated an increase in the level of OPN protein induced by IL-10 (10 ng/mL) in an IL-18 concentration-dependent manner (Figure S10 in Supplementary Material). These observations are supported by earlier studies showing the ability of IL-10 and IL-18 to increase OPN levels in human monocytes (56, 57). Of importance, an anti-OPN Ab strongly suppressed increases in CD163 expression (Figure 5E) and tube-like structures (Figure 5F) observed in Mφ (IL-10) and Mφ (IL-10 + IL-18) with little influence on CD206 expression (Figure S11 in Supplementary Material), while in Mφ (IL-10) and Mφ (IL-10 + IL-18) this Ab significantly or slightly increased the expression of CD54 or CD86, respectively (Figure S11 in Supplementary Material).

### Thrombin Contributes to Augmentation of Mφ M2 Polarization and Angiogenic Capacity through Proteolytic Modification for OPN

**Figure 6:**
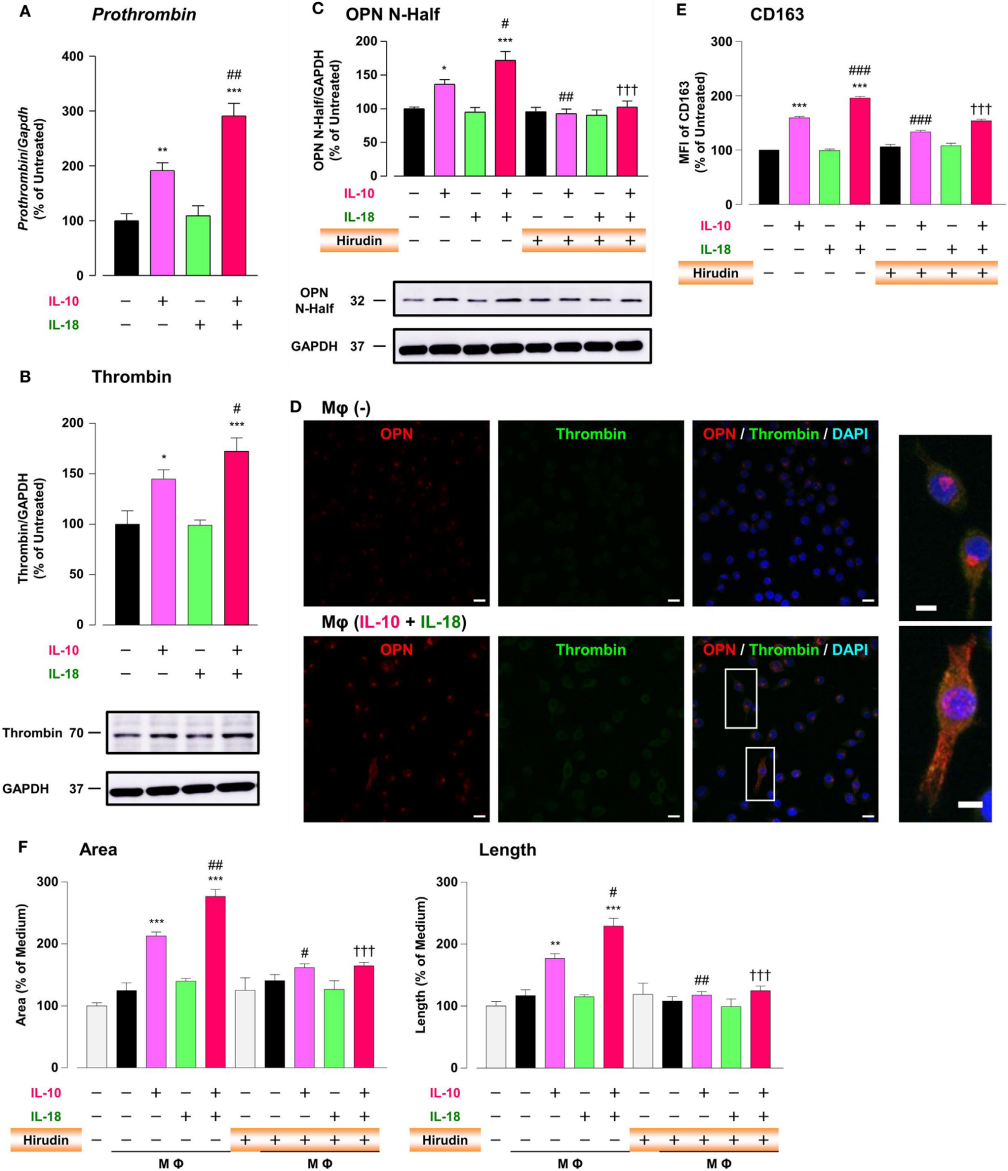
Thrombin contributes to macrophage (Mφ) M2 polarization and angiogenic capacity through proteolytic modification for osteopontin (OPN). **(A)** The mRNA expression level of *Prothrombin* relative to glyceraldehyde-3-phosphate dehydrogenase (*Gapdh*) was analyzed by reverse transcription polymerase chain reaction in each Mφ subset and were normalized to Mφ (–), *n* = 7 [***p < 0.001, **p < 0.01 vs. untreated, ^##^*p* < 0.01 vs. interleukin (IL)-10 alone]. **(B,C)** The protein expression levels of **(B)** thrombin or **(C)** OPN N-Half relative to GAPDH were measured by western blotting in each Mφ subset and were normalized to Mφ (–). Lower panels are typical images of each protein. **(B)** *n* = 8 (***p < 0.001, *p < 0.05 vs. untreated, ^#^*p* < 0.05 vs. IL-10 alone). **(C)** *n* = 16 (***p < 0.001, *p < 0.05 vs. untreated, ^##^*p* < 0.01, ^#^*p* < 0.05 vs. IL-10 alone, ^†††^*p* < 0.001 vs. IL-10 + IL-18). **(D)** Representative confocal laser scanning immunofluorescence images of OPN (red), thrombin (green), and their merge with DAPI (blue) in each Mφ subset. Scale bar represents 20 µm. Higher magnification images are from the white rectangle region in merged panel of Mφ (IL-10 + IL-18). Scale bar represents 10 µm. **(E)** Relative mean fluorescence intensity (MFI) of CD163 was measured by FACS analysis in each Mφ subset. Hirudin, a specific thrombin inhibitor, was used at 1 µg/mL, *n* = 3 (***p < 0.001 vs. untreated, ^###^*p* < 0.001 vs. IL-10 alone, ^†††^*p* < 0.001 vs. IL-10 + IL-18). **(F)** The total areas and lengths of tube-like structures were determined by the Matrigel tube formation assay where b.End5 were cocultured with each Mφ subset. Hirudin was used at 1 µg/mL, *n* = 6 (***p < 0.001, **p < 0.01 vs. untreated, ^##^*p* < 0.01, ^#^*p* < 0.05 vs. IL-10 alone, ^†††^*p* < 0.001 vs. IL-10 + IL-18). All data are expressed as means ± SEM and were analyzed by a one-way ANOVA followed by Tukey’s test.

It has long been recognized that there is a molecular crosstalk between the coagulation cascade and inflammatory pathways, although little information exists on the exact role of thrombin, the key terminal enzyme of coagulation system, in the inflammatory response. A growing body of evidence demonstrated that in RA and tumor micromilieu coagulation cascade is activated to amplify and perpetuate these diseases (12, 13, 58), and that increased levels of coagulation factors like thrombin contribute to several features of their pathogenesis including an abnormal angiogenic response (12, 13, 19). Furthermore, because thrombin cleaves OPN to generate Thr-OPN known as a highly active fragment of OPN (21), we assessed the role of thrombin as a key modulator for OPN activity. Significant increases in the *prothrombin* mRNA (Figure 6A), the protein expression of thrombin (Figure 6B; Figure S9B in Supplementary Material) and OPN N-Half (Thr-OPN), reflecting the N-terminal fragment of OPN cleaved by thrombin (Figure 6C; Figure S9C in Supplementary Material) were observed in Mφ (IL-10) those were greatly potentiated in Mφ (IL-10 + IL-18) (Figures 6A–C). Moreover, increased expressions of thrombin (green) and OPN (red) were highly co-localized in cytoplasm of Mφs (IL-10 + IL-18) accompanied by morphological alteration (Figure 6D). The present data are supported by the fact that the expression level and enzymatic activity of thrombin localized at Mφ were drastically elevated in response to inflammatory stimuli (59, 60). Because there is evidence that hirudin, a highly specific thrombin antagonist, ameliorated RA (19) and cancer progression (24), we next checked the influence of hirudin on Mφ polarization process and angiogenic potency. Interestingly, hirudin clearly suppressed increases in OPN N-Half expression (Figure 6C; Figure S9C in Supplementary Material) and the surface CD163 level (Figure 6E), as well as the magnitude of tube formation (Figure 6F) induced by Mφ (IL-10) or Mφ (IL-10 + IL-18), back to the control level, without any impact on other surface markers (Figure S12 in Supplementary Material). Another importance is that VEGF-dependent facilitation in the capillary-like network formation was not abolished by the use of hirudin (Figure S13 in Supplementary Material), emphasizing that Thr-OPN but not that receiving further cleavage by other proteases contributes to an increase in the Mφ surface expression of CD163, which leads to excessive angiogenesis. Thus, thrombin derived from Mφ may act in concert with OPN to generate Thr-OPN, potentiating M2 polarization and angiogenic activity of Mφ (Figure 1).

### Intergins α4/α9 Are Responsible for the Action of OPN in the Augmentation of Mφ M2 Polarization and Angiogenic Capacity

**Figure 7:**
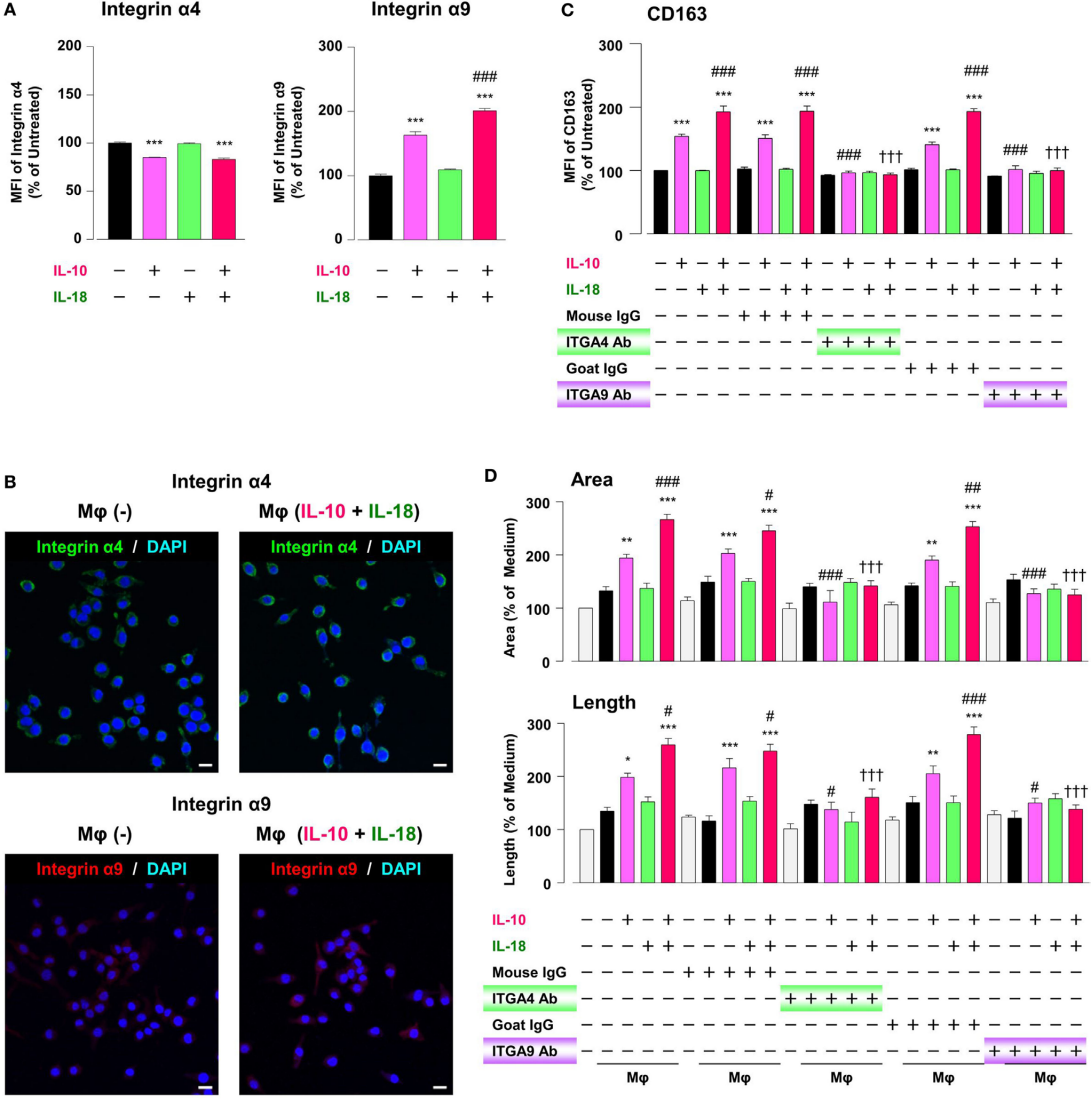
Intergins α4/α9 are responsible for the action of osteopontin (OPN) in the augmentation of macrophage (Mφ) M2 polarization and angiogenic capacity. **(A)** Relative mean fluorescence intensities (MFIs) of integrins α4/α9 were measured by FACS analysis in each Mφ subset. Anti-integrin α4 or α9 antibodies (Abs) and each isotype-matched control Ab were used at 10 µg/mL, *n* = 4 [***p < 0.001 vs. untreated, ^###^*p* < 0.001 vs. interleukin (IL)-10 alone]. **(B)** Representative confocal laser scanning immunofluorescence overlay images of integrin α4 (green) and DAPI (blue), as well as those of integrin α9 (red) and DAPI (blue) in Mφ (–) and Mφ (IL-10 + IL-18). Scale bar represents 20 µm. **(C)** Relative MFI of CD163 was measured by FACS analysis in each Mφ subset. Anti-integrin α4 or α9 Abs and each isotype-matched control Ab were used at 10 µg/mL, *n* = 4 (***p < 0.001 vs. untreated, ^###^*p* < 0.001 vs. IL-10 alone, ^†††^*p* < 0.001 vs. IL-10 + IL-18). **(D)** The total areas and lengths of tube-like structures were determined by the Matrigel tube formation assay where b.End5 was cocultured with each Mφ subset. Anti-integrin α4 or α9 Abs and each isotype-matched control Ab were used at 10 µg/mL, *n* = 6 (***p < 0.001, **p < 0.01, *p < 0.05 vs. untreated, ^###^*p* < 0.001, ^##^*p* < 0.01, ^#^*p* < 0.05 vs. IL-10 alone, ^†††^*p* < 0.001 vs. IL-10 + IL-18). Integrin α4 = ITGA4; Integrin α9 = ITGA9. All data are expressed as means ± SEM and were analyzed by a one-way ANOVA followed by Tukey’s test.

While intact OPN is recognized by various integrins, synovial and tumor Mφs express α4/α9 integrins abundantly, and both integrins are receptors for Thr-OPN (14, 61). We thereby turned to assess whether these integrins contribute to Mφ M2 polarization and angiogenic capacity. The surface α9 integrin expression in Mφ (IL-10) was considerably higher than that in Mφ (–), and this effect was significantly augmented in Mφ (IL-10 + IL-18) (Figure 7A). Conversely, α4 integrin level was equally decreased in Mφ (IL-10) and Mφ (IL-10 + IL-18) (Figure 7A), presumably due to the fact that α9 but not α4 integrin recognizes Thr-OPN with high specificity (61). Additionally, immunofluorescence analysis showed the similar results that α9 intergin expression was apparently increased in Mφ (IL-10 + IL-18) relative to Mφ (–), while there were little changes in α4 integrin expression between two groups (Figure 7B). Interestingly, facilitations of CD163 expression (Figure 7C) and tubulogenesis (Figure 7D) in Mφ (IL-10) and Mφ (IL-10 + IL-18) were drastically suppressed by neutralization of either α4 or α9 integrin to the level of Mφ (–). On the other hand, both Abs slightly increased CD54 and CD206 level (Figure S14 in Supplementary Material). Taken together, α4/α9 integrins are responsible for mediating Thr-OPN action to amplify M2 polarization and angiogenic capacity of Mφ (Figure 1).

### CD163 Is a Critical Factor for Determining the Angiogenic Capacity of Mφ

**Figure 8:**
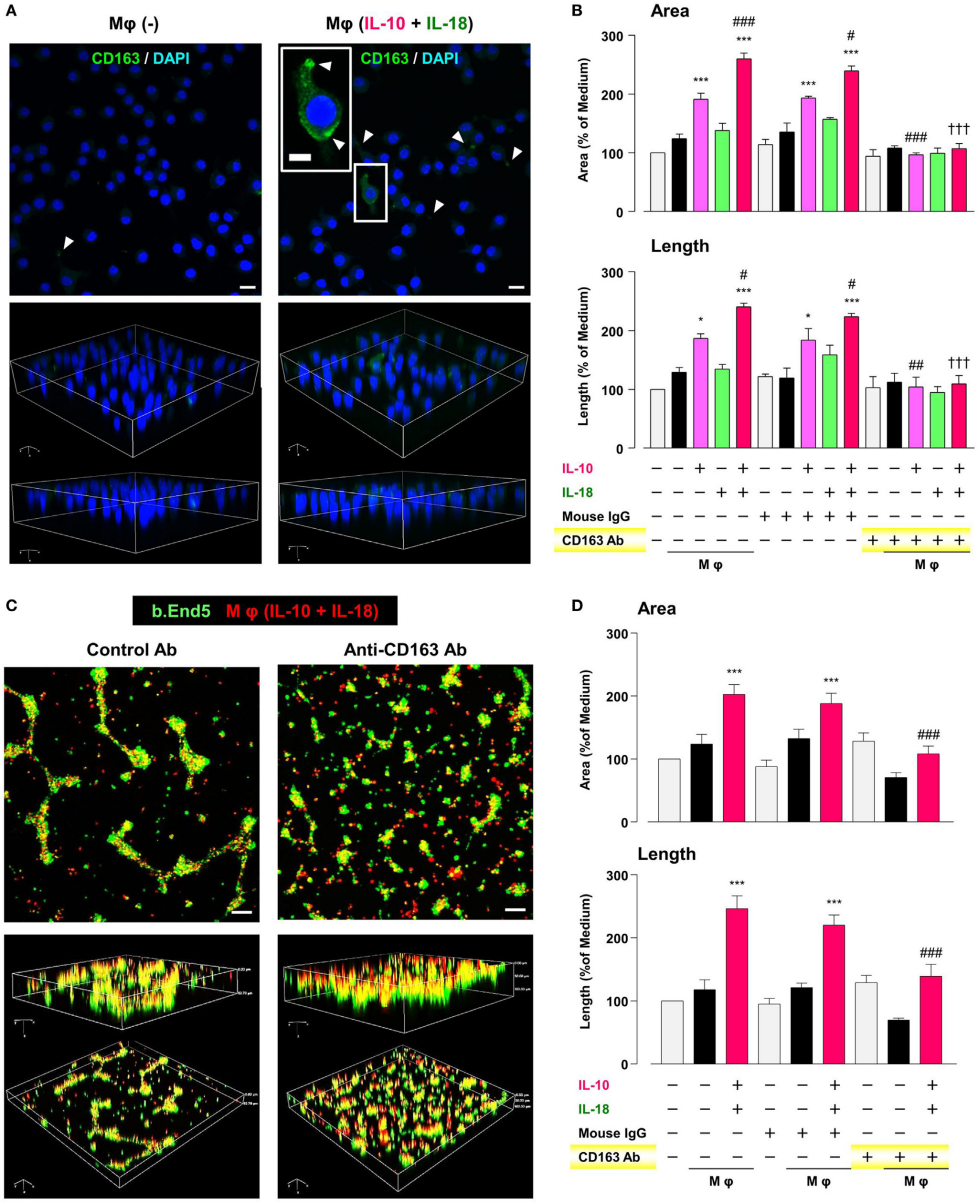
CD163 is a critical factor for determining the angiogenic capacity of macrophage (Mφ). **(A)** Upper; representative confocal laser scanning immunofluorescence overlay images of CD163 (green) and DAPI (blue) in Mφ (–) and Mφ [interleukin (IL)-10 + IL-18]. Scale bar represents 20 µm. Lower; Three-dimensional images of each upper panel. Higher magnification image in the panel of Mφ (IL-10 + IL-18) is from white rectangle region. Scale bar represents 10 µm. White arrowheads indicate CD163 highly expressed and localized at pseudopodia. **(B)** The total areas and lengths of tube-like structures were determined by the Matrigel tube formation assay where b.End5 were cocultured with each Mφ subset. An anti-CD163 antibody (Ab) and its isotype-matched control Ab were used at 4 µg/mL, *n* = 3 (***p < 0.001, *p < 0.05 vs. untreated, ^###^*p* < 0.001, ^##^*p* < 0.01, ^#^*p* < 0.05 vs. IL-10 alone, ^†††^*p* < 0.001 vs. IL-10 + IL-18). **(C)** Representative images (upper) and corresponding three-dimensional images (lower) of tube-like structures as well as **(D)** total areas and lengths of tubular structure where b.End5 (green) and Mφs (IL-10 + IL-18) (red) were cocultured on Matrigel for 16 h with an anti-CD163 Ab or its isotype-matched control Ab. Scale bar represents 100 µm, *n* = 8 (***p < 0.001 vs. untreated, ^###^*p* < 0.001 vs. IL-10 + IL-18). All data are expressed as means ± SEM and were analyzed by a one-way ANOVA followed by Tukey’s test.

CD163 has recently drawn attention to its positive correlation with microvessel density under some pathological conditions (62–64). Therefore, we finally asked if CD163 had any impact on the angiogenic capacity of Mφ (IL-10) and Mφ (IL-10 + IL-18). Confocal laser scanning immunofluorescence microscopy showed that fluorescence signal intensity of CD163 (green) was highly detected at the region of pseudopodia in Mφ (IL-10 + IL-18), but rarely found in Mφ (–) (Figure 8A). Neutralization of CD163 abolished the facilitation of tubulogenesis induced by Mφ (IL-10) and Mφ (IL-10 + IL-18) to the control level (Figure 8B). More importantly, this Ab also disrupted co-localization and/or cell–cell contact between b.End5 (green) and Mφ (IL-10 + IL-18) (red), resulting in a failure of vascularization (Figures 8C,D). Our findings suggest an important role of CD163 in mediating the direct cell–cell interaction between Mφs and endothelial cells during angiogenesis (Figure 1).

## Discussion

Macrophage is a crucial component of microenvironment and believed to be local and systemic amplifier of various chronic inflammatory diseases including RA and cancer in which excessive angiogenesis is a detrimental factor (65, 66). However, it is poorly understood how Mφs contribute to the development and persistence of these pathophysiology. Therefore, to provide a model widely applicable for the drug development, it is necessary to establish a simplified experimental method to assess the impact of microenvironment in various chronic inflammatory diseases on several aspects of Mφ including polarization status, morphology, and functional activity.

Our results identified IL-18 as a selective amplifier for an increase in CD163 level exhibited by IL-10, that is, the aug-mentation of M2 polarization (Figures 2A,B; Figure S2 in Supplementary Material). Furthermore, IL-18 also facilitated angiogenic potency of Mφ (IL-10) with higher expression of CD163 (Figures 2E,F), which partly illustrates the mechanism underlying the role of IL-18 in excessive angiogenesis within micromilieu. As demonstrated by Henan et al., stimulation of IL-18 for 5 days enhanced the phagocytosis of RAW264.7 cells and this Mφ also damaged tumor endothelial cells (67). By contrast, in this study, no changes were observed in the phagocytic activity and the cytotoxicity against endothelial cells by treatment of RAW264.7 cells with IL-18 alone or in combination with IL-10 for 24 h (Figures S6A,B in Supplementary Material). This is probably due to a difference in the stimulation period of IL-18. Therefore, we succeed to develop the *in vitro* experimental model that mimics micromilieu where IL-18 induces excessive angiogenesis by potentiating Mφ M2 polarization (Figure 1). Of particular interest, IL-33, a recently identified member of the IL-1 family cytokine (68), oriented Mφ toward both M1/M2 phenotypes *per se*, but had little influence on the action of IL-10, while IL-13 alone and in combination with IL-10 both amplified Mφ M2 polarization (Figure S15 in Supplementary Material). Given that IL-13 and IL-33 exhibited considerably different characters from IL-18, further studies are required to understand the influence of these cytokines on the polarization status and angiogenic capacity of Mφ.

During angiogenesis, Mφs were identified in the perivascular space outside the basement membrane (69, 70). Interestingly, pharmacological depletion of Mφs suppressed normal vascular development and pathological neovascularization (71, 72). However, the precise kinetics and dynamics with regard to temporal behavior and actions of Mφs as well as its interaction with endothelia during angiogenesis remains to be determined. By means of live-cell imaging (Figure 3; Figure S7 and Videos S1–S5 in Supplementary Material) and morphological analysis (Figure 4; Figure S8 in Supplementary Material), we demonstrated, for the first time, that M2-like Mφs serve not only as carriers for endothelial cells to the capillaries at which vascular sprouting and/or junction occur but also as bridge between the neighboring endothelial cells mediated *via* direct cell–cell interaction, eventually growing vascular network. In the *in vivo* studies, tissue resident Mφ also accelerates vascular development by bridging the neighboring tip cells and by mediating anastomosis of adjacent capillary tubes (53, 54). Together, these observations propose a potential importance of direct cell–cell interaction between M2-like Mφs and endothelia during excessive angiogenic induction.

To explore the mechanism by which IL-18 augments IL-10-induced M2 polarization and angiogenic potency of Mφ, we focused on OPN according to the results of a comprehensive protein analysis (Figure 5A). OPN undergoes posttranslational processing by several proteases including thrombin (21), hereby generates the N-terminal fragment of OPN cleaved by thrombin (Thr-OPN) that binds selectively to α4/α9 integrins (61). As is the case in our experimental model (Figures 5A–D and 6A–D) OPN, thrombin, and Thr-OPN are all detected at higher levels in the microenvironments of RA (12, 22, 73) and tumor (58, 74, 75). In addition, synovial and tumor Mφs express α4/α9 integrins abundantly (14, 61) in analogy with Figures 7A,B. Recent studies have indicated that OPN not only skews Mφ toward M2-like phenotype (52, 76) but also downregulates M1 Mφ marker levels (77, 78), although OPN was originally identified as an inflammatory cytokine (79). This is probably due to the complexity of OPN existing as a variety of cleaved forms by several proteases processing, each of which act through different integrin receptors, and to the heterogeneous nature of Mφs themselves with distinct OPN receptors under several pathological conditions (80). Intriguingly, OPN has attracted considerable attention as an exacerbating factor for pathogenesis of RA and cancer by promoting angiogenesis (17, 18). Moreover, there is evidence for antagonism of either thrombin (19, 24), α4 integrin (81, 82), or α9 integrin (22, 83) yielding remarkable therapeutic effects against RA and cancer. In fact, facilitations of CD163 expression and angiogenic activity in Mφ (IL-10) and Mφ (IL-10 + IL-18) were mediated through OPN (Figures 5E,F), thrombin (Figures 6E,F), and α4/α9 integrins (Figures 7C,D). Thus, IL-18 amplifies the production of OPN and thrombin as soluble mediators derived from Mφ, leading to Thr-OPN generation, which drives M2 polarization through α4/α9 integrins, ultimately resulting in excessive angiogenesis (Figure 1). Our present data might provide insight at the molecular level into why the coagulation system centering on thrombin is upregulated and how this system contributes to excessive angiogenesis under some chronic inflammatory conditions, including RA and cancer (12, 13).

CD163 was first identified and most studied as a functional receptor to bind and internalize hemoglobin–haptoglobin complexes (84). Later, its functional repertoire was expanded and significant associations of microvessel density with CD163 expression were demonstrated in several pathological conditions such as cancers (62, 63). Additionally, CD163-positive Mφs were highly accumulated in foci of RA (43, 85) and cancer patients (86, 87); however, their implication for the pathogenesis has yet to be remained. We discovered that in Mφ (IL-10 + IL-18) CD163 was increased in the surface membrane (Figures 2A,B), and in particular, highly localized at the region of pseudopodia in association with morphological alteration (Figure 8A). Interestingly, CD163 contributed to angiogenic potency of Mφ (IL-10 + IL-18) (Figure 8B) through the direct cell–cell interaction with endothelium (Figures 8C,D). Although concentrations of soluble CD163 (sCD163) were several-fold higher in patients with RA (88) and some hematologic malignancy (89, 90), sCD163 level in the culture medium of Mφs were undetectable in our experimental system (data not shown). These observations imply that CD163 present as surface membrane-bound form but not as soluble form may be responsible for driving the cell–cell interaction between Mφ (IL-10 + IL-18) and endothelium, leading to excessive angiogenesis (Figure 1). Taking into account the existence of various cleaved forms of OPN, a therapeutic strategy targeting for CD163 might be easier and more fruitful than that for OPN. Therefore, further research is required to understand how CD163 modulates the cell–cell interaction between endothelial cells and Mφs to induce angiogenesis.

Accumulated evidence suggests that numerous pro-angiogenic mediators, such as VEGF, serve as key regulators for accelerating angiogenesis in RA (91) and cancer foci (92). VEGF is produced and released in response to pro-inflammatory cytokines, such as TNF-α and IL-18 within synovial micromilieu in which perivascular and synovial fibroblasts are primary sources of VEGF (91), supporting our results that the expression levels of VEGF and its receptors in Mφ (IL-10), Mφ (IL-18), and Mφ (IL-10 + IL-18) were similar to those in Mφ (–) (Figure S16A in Supplementary Material) and that an anti-VEGF Ab never had significant influences on increases in the surface expressions of CD163 and angiogenic capacity observed in Mφ (IL-10) and Mφ (IL-10 + IL-18) (Figures S16B,C in Supplementary Material).

On the other hand, OPN is cleaved not only by thrombin but also by matrix metalloproteinase (MMP)-2/3/7/9 (93), among which MMP-3/7-cleaved form of OPN acts as a ligand for α9 integrin (94). The expression level or enzymatic activity of MMPs-2/3/7/9 were elevated in microenvironment of RA and cancer (64, 95, 96); however, the exact role of MMPs under these pathological conditions has not yet been determined, even to the extent whether these proteinases behave as enhancers or suppressors in the progression of these diseases (96). In fact, the mRNA levels of *Mmp*-*2*/*3*/*7*/*9* in Mφ (IL-10) and Mφ (IL-10 + IL-18) were similar to those in Mφ (–), while *Mmp*-*2*/*9* mRNA levels were greatly increased in Mφ (TNF-α) relative to Mφ (–) (Figure S17 in Supplementary Material), suggesting there is no involvement of MMPs in the actions of IL-10 and IL-18. Together, VEGF and MMPs detected at higher levels in the RA and tumor foci may be derived from endothelial cells or fibroblasts and all that other than Mφs among components of the micromilieu.

Other importance is that M-CSF has been classified as an M2 stimulus and produces some growth factors, such as VEGF (2, 97). Intriguingly, M-CSF is required for pathological angiogenesis in tumors (69). In fact, numerous studies have revealed that the expression of M-CSF in tumor microenvironment is closely correlated with the number of CD163-positive M2 Mφs, which in turn contributes to the progression of several cancers, most likely by enhancing angiogenesis (97–99). By contrast, both the protein expressions of M-CSF and M-CSF1R were decreased not only in Mφ (IL-10) and Mφ (IL-10 + IL-18) but also in Mφ (TNF-α) and Mφ (TNF-α + IL-18) (Figure S18A in Supplementary Material). This discrepancy may be attributed to the different situation where Mφs exist as single or with tumor cells or endothelial cells, although an elevation of M-CSF in M2 Mφs is undoubtedly involved in the excessive angiogenesis. In addition, anti-M-CSF Ab had little impacts on increases in the surface expressions of CD163 and angiogenic capacity observed in Mφ (IL-10) and Mφ (IL-10 + IL-18) (Figures S18B,C in Supplementary Material). Thus, M-CSF and M-CSF1R might not be involved in the mechanism by which IL-18 augments M2 polarization and angiogenic capacity of Mφ.

In conclusion, we demonstrated that IL-18 with IL-10 synergistically amplifies the expression of OPN and thrombin as soluble mediators derived from Mφ, yielding Thr-OPN generation which acts through α4/α9 integrins, ultimately augment M2 polarization of Mφ with characteristics of increasing CD163 expression. These Mφs strongly induced angiogenesis by modulating the direct contact with endothelium, highlighting a potential role of CD163 in the excessive angiogenesis probably through the direct cell–cell interaction between M2-like Mφs and endothelial cells (Figure 1). Importantly, we found, for the first time, that the characteristic behavior and action of Mφ as well as its morphological alteration during angiogenesis using the live-cell imaging model and SEM analysis. To search for the therapeutic targets responsible for various chronic inflammatory diseases which share a common characteristic of excessive angiogenesis, future research should provide cellular insights that would help to illustrate the mechanism by which M2-like Mφs contribute to excessive angiogenesis *via* CD163.

## Author Contributions

Conceptualization: HT; methodology: TK, SH, HW, SM, and HT; formal analysis: TN and AN; investigation: TK and YY; resources: SH, AK, SN, TY, and MN; writing-original draft: TK; writing, review, and editing: TK, MN, and HT; visualization: TK, YY, and TN; supervision: SN, SM, TY, MN, and HT; funding acquisition: TK, AK, AN, MN, and HT.

## Conflict of Interest Statement

The authors declare that the research was conducted in the absence of any commercial or financial relationships that could be construed as a potential conflict of interest.
