# Supplementary material for: Interleukin-18 Amplifies Macrophage Polarization and Morphological Alteration, Leading to Excessive Angiogenesis
Source: Front Immunol. 2018 Mar 6;9:334. doi: 10.3389/fimmu.2018.00334 (PMC5845536; doi:10.3389/fimmu.2018.00334)
Supplement: Supplementary file 6 [file data_sheet_1.PDF]

Supplementary Material

Interleukin-18 Amplifies Macrophage Polarization and Morphological Alteration, Leading to Excessive Angiogenesis.

Takuro Kobori, Shinichi Hamasaki, Atsuhiro Kitaura, Yui Yamazaki, Takashi Nishinaka, Atsuko Niwa, Shinichi Nakao, Hidenori Wake, Shuji Mori, Tadashi Yoshino, Masahiro Nishibori, and Hideo Takahashi*

***Correspondence:**
Hideo Takahashi
hkt@med.kindai.ac.jp

Supplementary Figures and Videos

Supplementary Figures

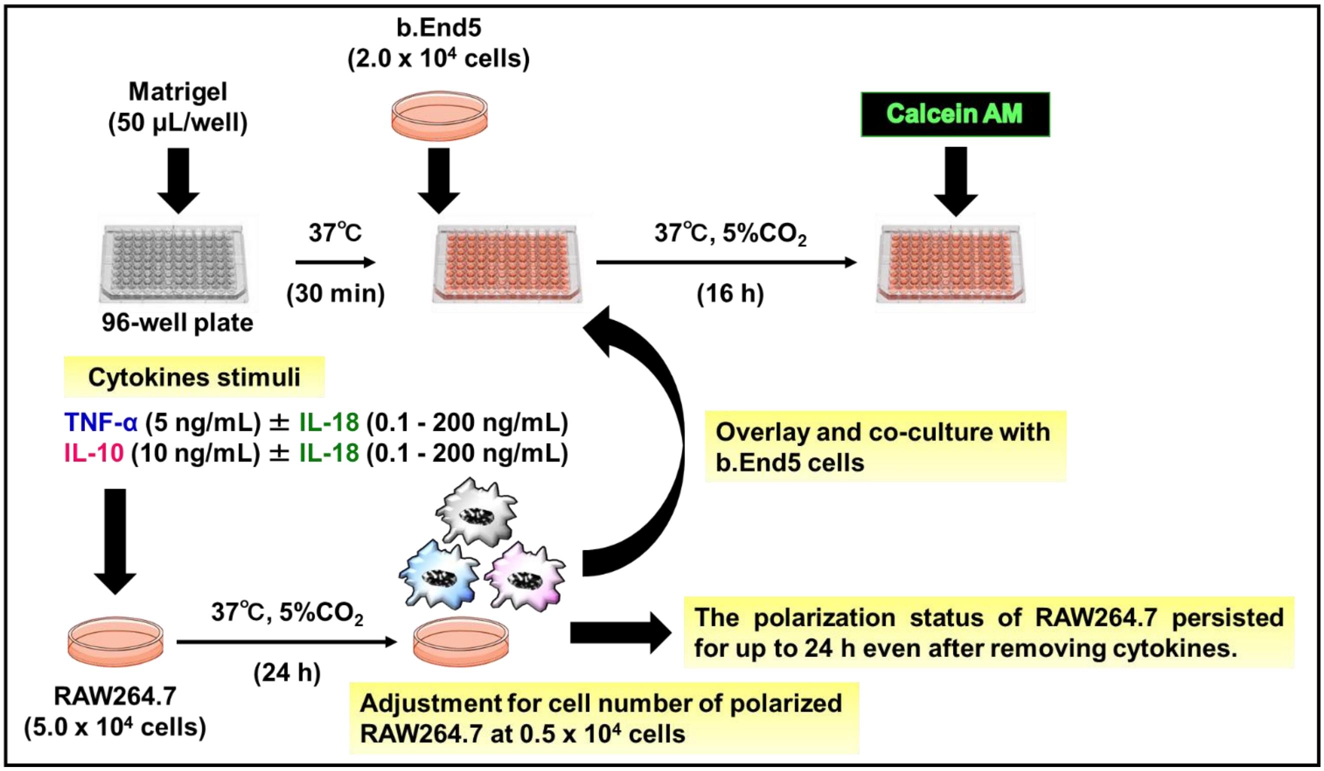

Supplementary Figure 1. Simplified Experimental Protocol for the Matrigel Tube Formation Assay. Ninety-six-well plates were filled with 50 μL of Matrigel and allowed to solidify at 37°C for 30 min after which b.End5 cells (2.0 × 10^4 cells/100 μL) were gently seeded on top of the gel. Subsequently, RAW264.7 cells polarized by tumor necrosis factor (TNF)-α (5 ng/mL) or interleukin (IL)-10 (10 ng/mL) in the presence or absence of IL-18 (0.1 to 200 ng/mL) for 24 h at 37°C under 5% CO_2, were rinsed 3 times with phosphate-buffered saline followed by adjustment of the cell number to 0.5 × 10^4/100 μL. Polarized RAW264.7 cells were then co-cultured with b.End5 cells on Matrigel for 16 h at 37°C under 5% CO_2. After incubation, tube-like structures were visualized by calcein acetoxymethylester (AM) staining, which allowed to calculate and evaluate their areas and total lengths as the degree of tube formation.

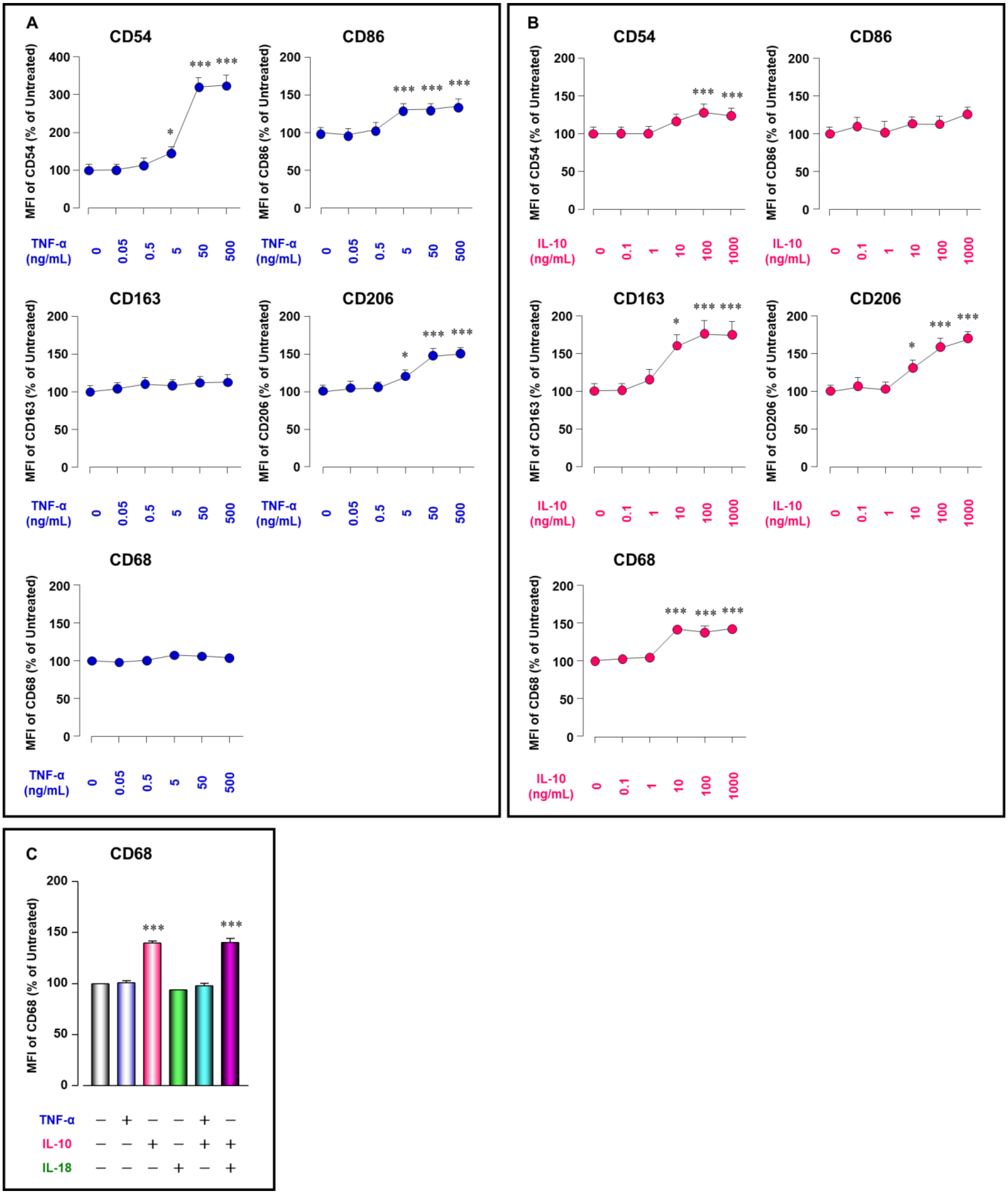
A
CD54
CD86
CD163
CD206
CD68
MFI of CD54 (% of Untreated)
MFI of CD86 (% of Untreated)
MFI of CD163 (% of Untreated)
MFI of CD206 (% of Untreated)
MFI of CD68 (% of Untreated)
TNF-α (ng/mL)
0 0.05 0.5 5 50 500
B
CD54
CD86
CD163
CD206
CD68
IL-10 (ng/mL)
0 0.1 1 10 100 1000
C
CD68
MFI of CD68 (% of Untreated)
TNF-α − + − − + −
IL-10 − − + − + +
IL-18 − − − + − +

Supplementary Figure 2. **Concentration-dependent Effects of Cytokine Stimuli on Macrophage (Mφ) Surface Markers. (A-B)** After incubating RAW264.7 cells with tumor necrosis factor (TNF)-α (0.05-500 ng/mL) or interleukin (IL)-10 (0.1-1,000 ng/mL) for 24 h at 37°C under 5% CO_2, the mean fluorescent intensities (MFIs) of CD54, CD86, CD163, CD206, CD68, and Mφ colony-stimulating factor-1 receptor (M-CSF1R) on the surface membrane were determined by FACS analysis. (A) Treatment with TNF-α concentration-dependently increased the expression levels of CD54, CD86, and CD206 with little changes in CD163 and CD68 levels in a concentration-dependent manner. (B) Treatment with IL-10 concentration-dependently increased the expression levels of CD163, CD206, and CD68 without a significant change in the level of CD86 in a concentration-dependent manner. Note, however, that IL-10 used at high concentrations (100 or 1,000 ng/mL) increased the levels of CD54 moderately. Therefore in subsequent experiments, we used TNF-α or IL-10 at concentrations of 5 or 10 ng/mL, respectively, to selectively, to some extent, induce the M1 or M2 Mφ phenotypes. n = 3-4. Data are expressed as means ± SEM and were analyzed by a one-way ANOVA followed by Dunnett's test. $^{***}p < 0.001$, $^{*}p < 0.05$ vs. untreated. **(C)** After incubating RAW264.7 cells with either TNF-α (5 ng/mL) and IL-10 (10 ng/mL) alone or in combination with IL-18 (100 ng/mL) for 24 h at 37°C under 5% CO_2, the MFI of CD68 on the surface membrane was determined by FACS analysis. IL-18 (100 ng/mL) never potentiated an increase in CD68 expression induced by IL-10 (10 ng/mL). n = 4. Data are expressed as means ± SEM and were analyzed by a one-way ANOVA followed by Tukey's test. $^{***}p < 0.001$ vs. untreated.

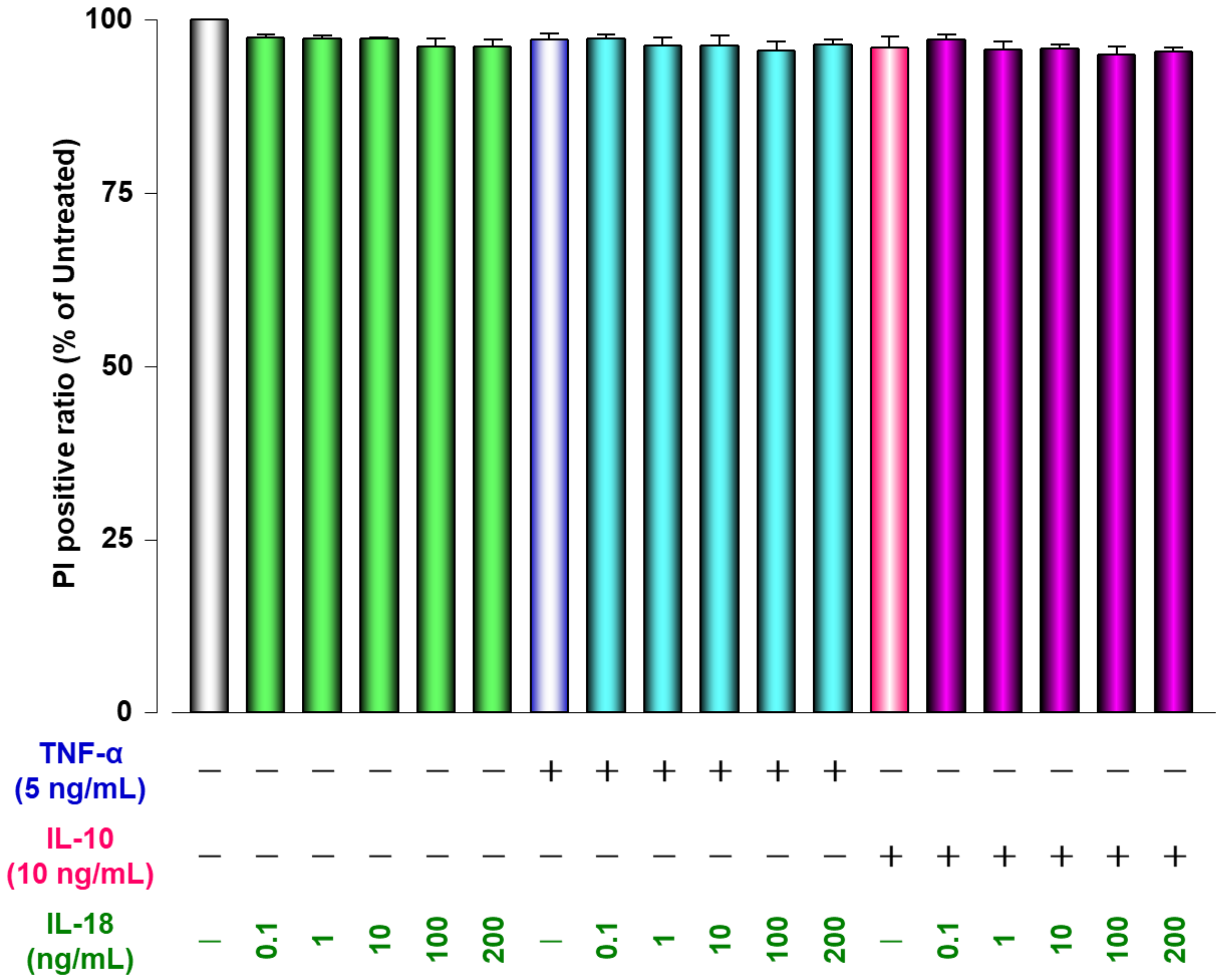

Supplementary Figure 3. Effect of Cytokine Stimuli on Macrophage (Mφ) Viability. After incubating RAW264.7 cells with tumor necrosis factor (TNF)-α (5 ng/mL), interleukin (IL)-10 (10 ng/mL), or IL-18 (0.1-200 ng/mL) alone or in combination for 24 h at 37°C under 5% CO_2, the ratio of propidium iodide (PI) positive cells per total cells was determined by FACS analysis. Stimulation of RAW264.7 cells by TNF-α (5 ng/mL), IL-10 (10 ng/mL), or IL-18 (0.1 to 200 ng/mL) each alone or their combination use had no obvious influence on the ratio of PI positive cells when compared with untreated cells. n = 5. Data are expressed as means ± SEM. There were no significant differences ($p > 0.05$).

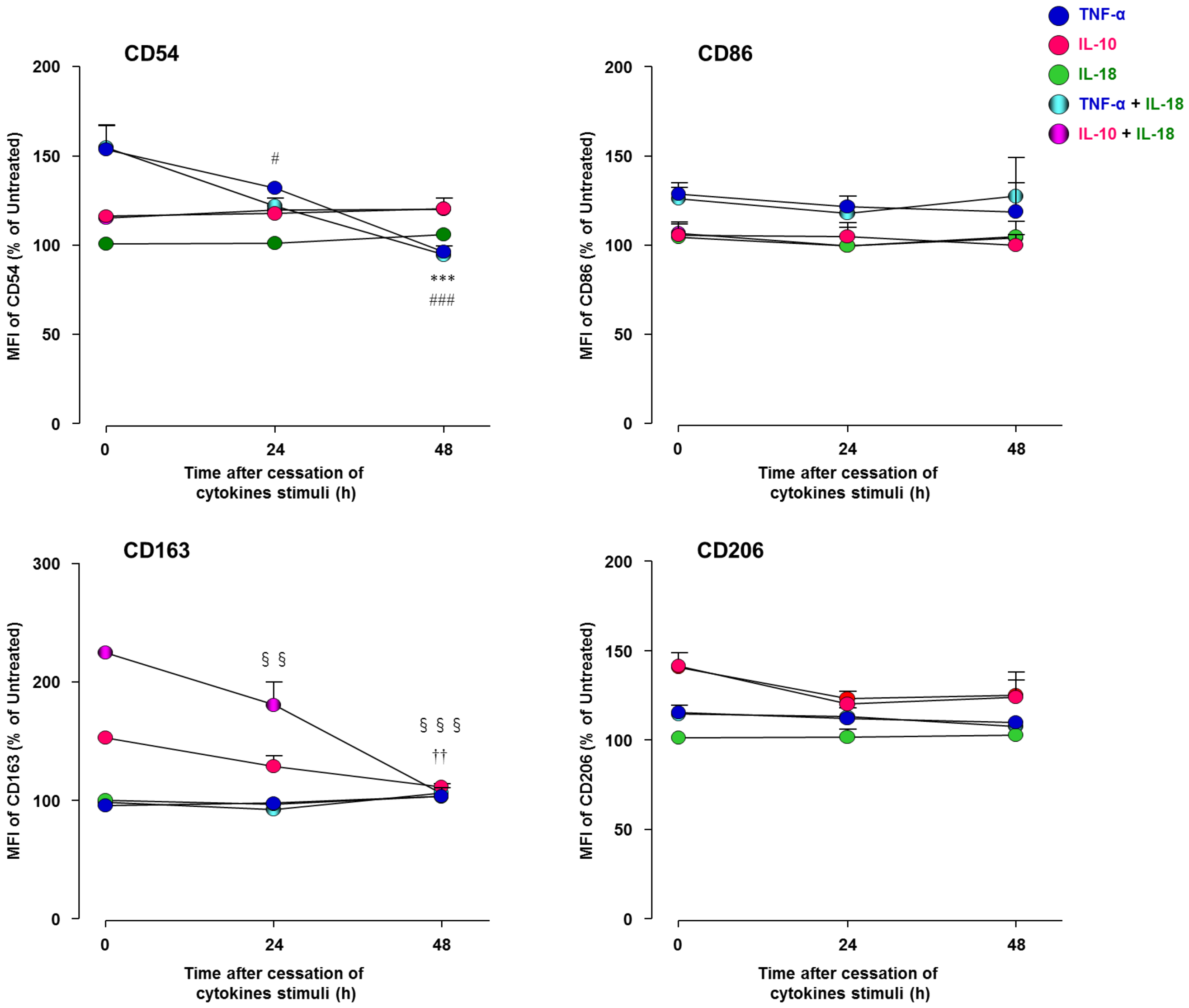

Supplementary Figure 4. Duration of Characteristic Macrophage (Mφ) Polarization Features Induced by Cytokine Treatment. After incubating RAW264.7 cells with tumor necrosis factor (TNF)-α (5 ng/mL) or interleukin (IL)-10 (10 ng/mL) in the presence or absence of IL-18 (100 ng/mL) for 24 h at 37°C under 5% CO_2, cells were rinsed with phosphate-buffered saline to remove cytokines. Subsequently, cells were supplemented with fresh medium followed by incubation for 0, 24, or 48 h at 37°C under 5% CO_2. The mean fluorescent intensities (MFIs) of CD54, CD86, and CD206 on the surface of RAW264.7 cells were measured by FACS analysis. Significant changes in the expression levels of Mφ M1/M2 markers persisted up to 24 h after cessation of exposure to cytokines, but declined to control levels at 48 h. Therefore, Mφ polarization status induced by cytokines persisted into the time when they were co-cultured with b.End5 cells on Matrigel. n = 4. Data are expressed as means ± SEM and were analyzed by a one-way ANOVA followed by Tukey's test. $^{***}p < 0.001$ vs. TNF-α (0 h), $^{\#\#\#}p < 0.001$, $^{\#}p < 0.05$ vs. TNF-α + IL-18 (0 h), $^{\dagger\dagger}p < 0.01$ vs. IL-10 (0 h), $^{\S\S\S}p < 0.001$, $^{\S\S}p < 0.01$ vs. IL-10 + IL-18 (0 h).

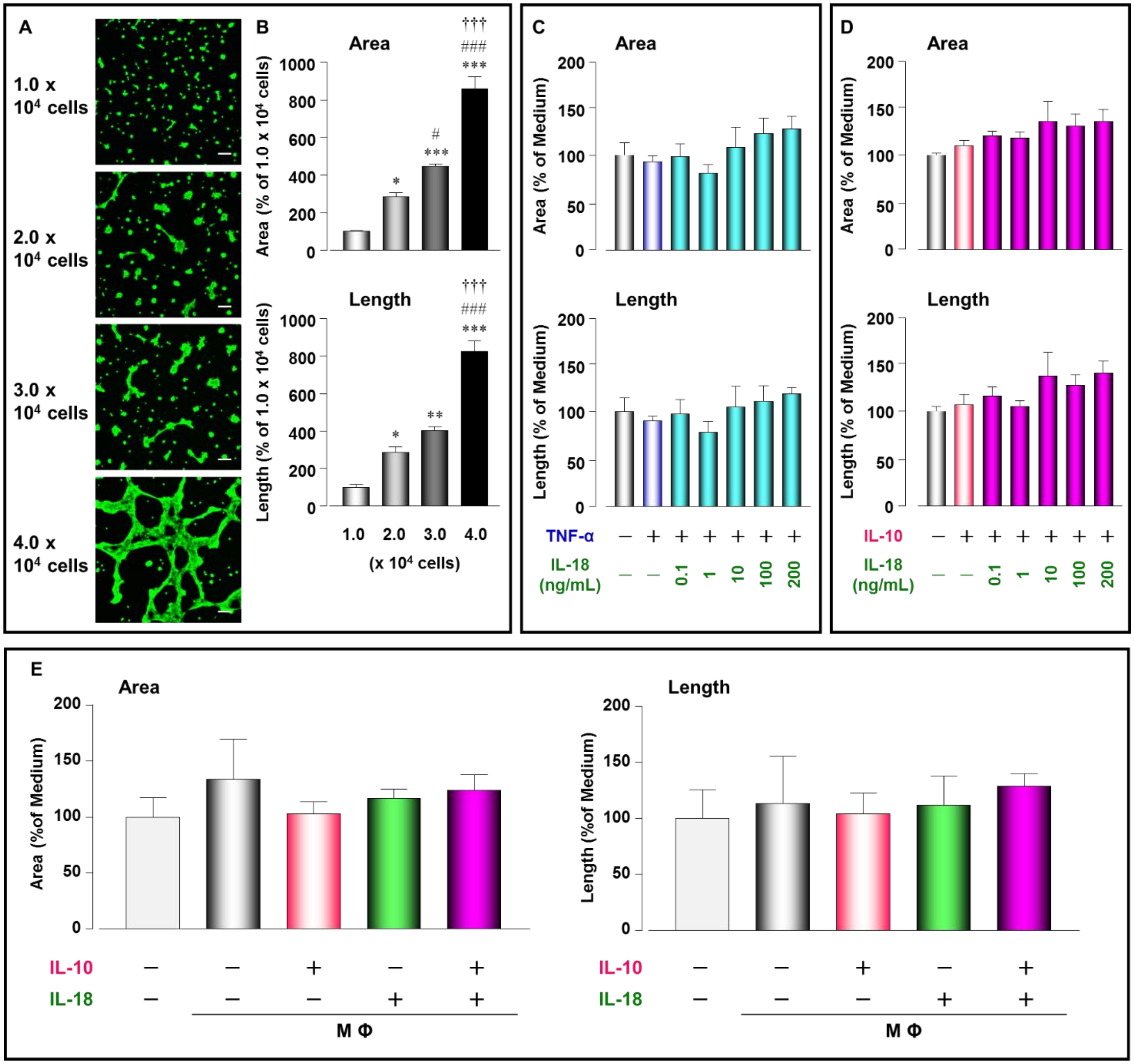

Supplementary Figure 5. **Establishment of *in vitro* Matrigel Tube Formation Assay. (A, B)** The b.End5 cell number dependent changes in the degree of tube formation. (A) Representative pictures of tube-like structures after culturing 1.0 × 10^4, 2.0 × 10^4, 3.0 × 10^4, or 4.0 × 10^4 cells of b.End5 cells on Matrigel for 16 h at 37°C under 5% CO_2. (B) The total areas and lengths of tube-like structures were calculated from each picture. The degree of tube formation was dependent upon the number of b.End5 cells from 1.0-4.0 × 10^4 cells seeded alone on Matrigel. Based on this result, subsequent experiments used 2.0 × 10^4 cells of b.End5 cells /well to detect enhanced tube formation. n = 4. Scale bars represent 100 μm. All data are expressed as means ± SEM and were analyzed by a one-way ANOVA followed by Tukey's test. $^{***}p < 0.001$, $^{**}p < 0.01$, $^{*}p < 0.05$ vs. 1.0 × 10^4 cells, $^{\#\#\#}p < 0.001$, $^{\#}p < 0.05$ vs. 2.0 × 10^4 cells, $^{\dagger\dagger\dagger}p < 0.001$ vs. 3.0 × 10^4 cells. **(C, D)** Changes in the degree of tube

formation by direct cytokine stimuli. After incubating b.End5 cells with tumor necrosis factor (TNF)-α (5 ng/mL) or interleukin (IL)-10 (10 ng/mL) in the presence or absence of IL-18 (0.1-200 ng/mL) for 16 h at 37°C under 5% CO_2, the total areas and lengths of tube-like structures were calculated from staining intensity of calcein acetoxymethylester. Direct treatment of b.End5 cells with (C) TNF-α (5 ng/mL) or (D) IL-10 (10 ng/mL) alone or each combined with IL-18 (0.1-200 ng/mL) had no effect on the formation of tube-like structures. Thus, there was no direct impact on tube formation even if the cytokines used for macrophage (Mφ) polarization before co-culturing with b.End5 cells remained in the culture medium. n = 4. Data are expressed as means ± SEM. There were no significant differences ($p > 0.05$). **(E)** Influence of secretory mediators derived from Mφs on the formation of tube-like network. After seeding b.End5 cells on top of Matrigel in the lower chamber, Mφ (-), Mφ (IL-10), Mφ (IL-18), or Mφ (IL-10 + IL-18) were seeded on the upper chamber of the transwell inserts after which they were indirectly co-cultured for 16 h at 37°C under 5% CO_2. Thereafter, the total areas and lengths of tube-like structures were calculated from staining intensity of calcein AM. None of media derived from each Mφ subset influenced the degree of tube formation in comparison with the medium only group where b.End5 cells were cultured in the absence of Mφs-derived soluble mediators, implying a crucial role of the direct cell-cell contact between Mφs and endothelial cells in facilitation of the angiogenesis. n = 3. Data are expressed as means ± SEM. There were no significant differences ($p > 0.05$).

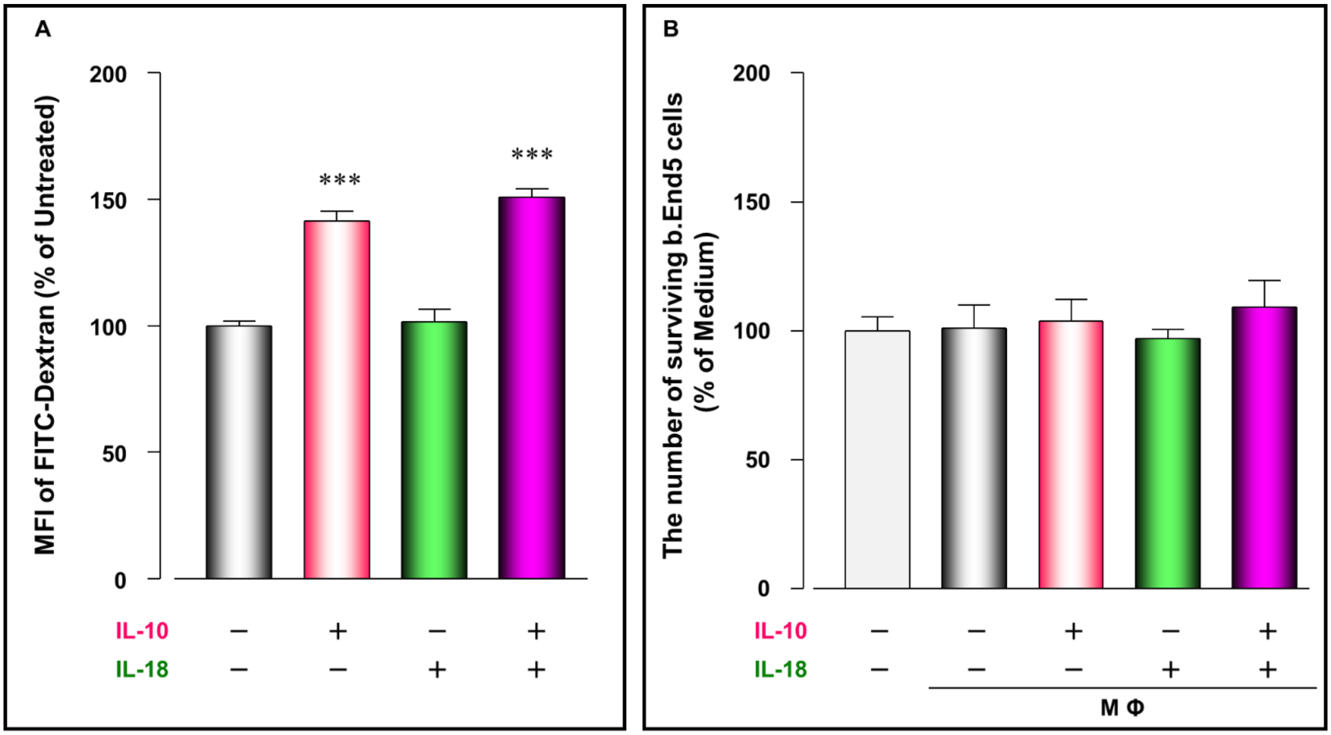

Supplementary Figure 6. **Changes in the Phagocytic Activity of Macrophages (Mφs) and the Cytotoxicity of Mφs against Endothelial Cells. (A)** Changes in the phagocytic activity of each Mφ subset. After incubating RAW264.7 cells with interleukin (IL)-10 (10 ng/mL) and IL-18 (100 ng/mL) either alone or their combination for 24 h at 37°C under 5% CO_2, the mean fluorescence intensity (MFI) of fluorescein isothiocyanate (FITC)-labeled dextran which is phagocytosed by each Mφ subset was measured by FACS analysis. The uptake level of dextran in Mφ (IL-10) was significantly higher than that in Mφ (-), while concomitant treatment with IL-18 never amplified the enhancement of dextran phagocytic activity in Mφ (IL-10). n = 8. Data are expressed as means ± SEM and were analyzed by a one-way ANOVA followed by Tukey's test. $^{***}p < 0.001$ vs. Mφ (-). **(B)** Changes in the survival ratio of b.End5 cells by co-culturing with each Mφ subset. After adhering PKH67 (green)-labeled b.End5 cells to the 96-well plate, RAW264.7 cells polarized with IL-10 (10 ng/mL) and IL-18 (100 ng/mL) alone or their combination for 24 h at 37°C under 5% CO_2 were overlaid on the fluorescent b.End5 cells, and subsequently co-cultured for 16 h at 37°C under 5% CO_2. In comparison with Mφ (-), none of Mφ subset had obvious influence on the area of PKH67 positive, which reflects the number of surviving b.End5 cells. n = 8. Data are expressed as means ± SEM. There were no significant differences ($p > 0.05$).

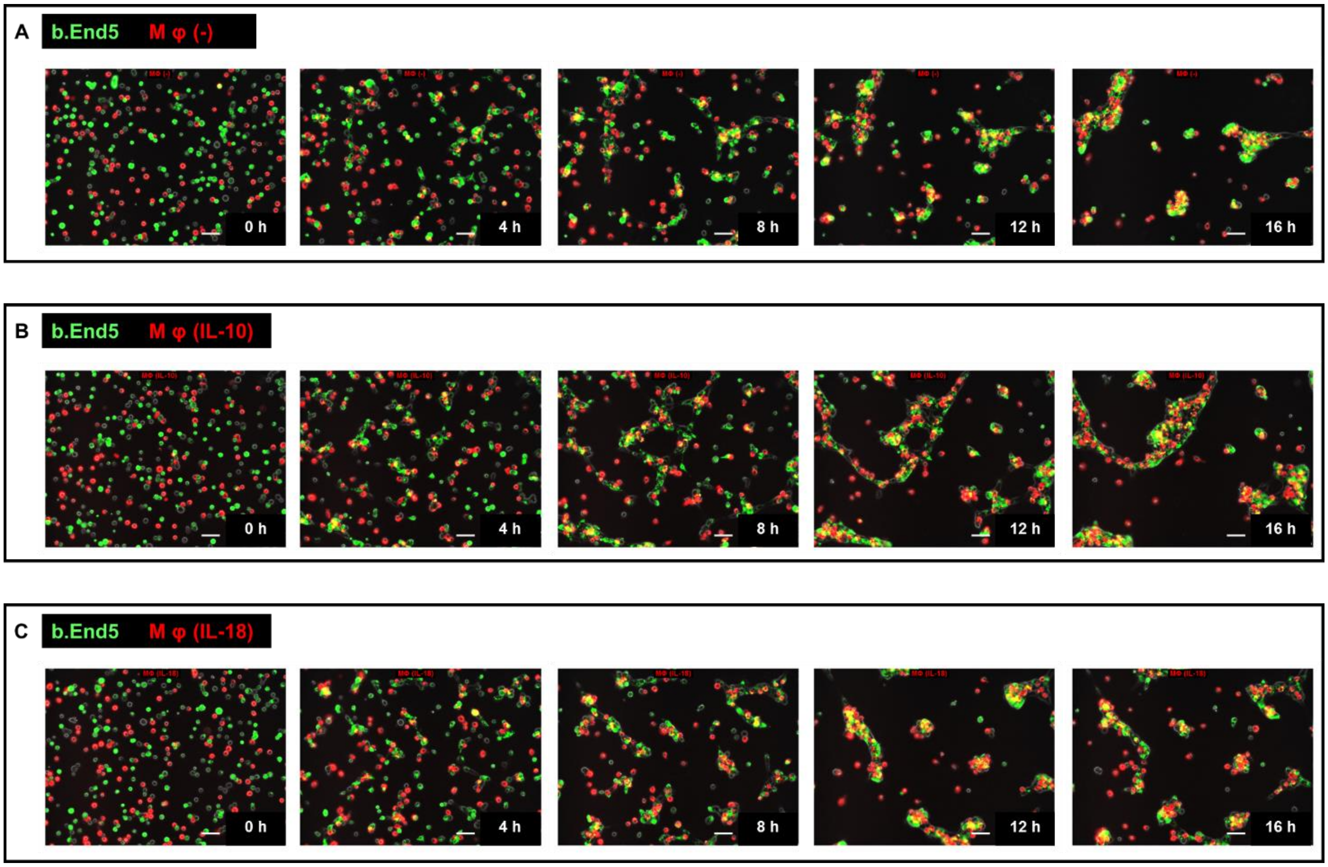

Supplementary Figure 7. **Temporal Behavior of Macrophage (Mφ) (-), Mφ [interleukin (IL)-10], and Mφ (IL-18) during Angiogenesis.** Representative series of time-lapse pictures in **(A)** Mφ (-), **(B)** Mφ (IL-10), or **(C)** Mφ (IL-18) were reconstructed from Videos S2, S3, or S4 in Supplementary Material, respectively, at 4 h intervals from 0 h to 16 h in the live-cell imaging of the Matrigel tube formation assay where endothelial cells (green) and each Mφ subset (red) were co-cultured. After starting the observation from 0 h to 3 h, PKH67 (green)-labeled b.End5 was in contact with each other from far and wide. Subsequently, Mφ (-) and Mφ (IL-18) hardly move or communicate with endothelial cells in comparison with the characteristic behavior of Mφ (IL-10 + IL-18) (**Figure 3,** and Video S1 in Supplementary Material). Intriguingly, vascular segments consists of b.End5 connected to each other through the help of PKH26 (red)-labeled Mφ (IL-10) mediated via direct cell-cell interaction from 3 h to 8 h, driving a rapid induction of tubulogenesis, as seen in Mφ (IL-10 + IL-18). Finally, a large number of each Mφ subsets gathered around the leading edge of the growing vascular network and/or branching points of vasculature where they interacted with endothelium, allowing vascular tube to get thicker and thicker as is the case with the behavior of Mφ (IL-10 + IL-18) (**Figure 3,** and Video S1 in Supplementary Material). Scale bar represents 50 μm.

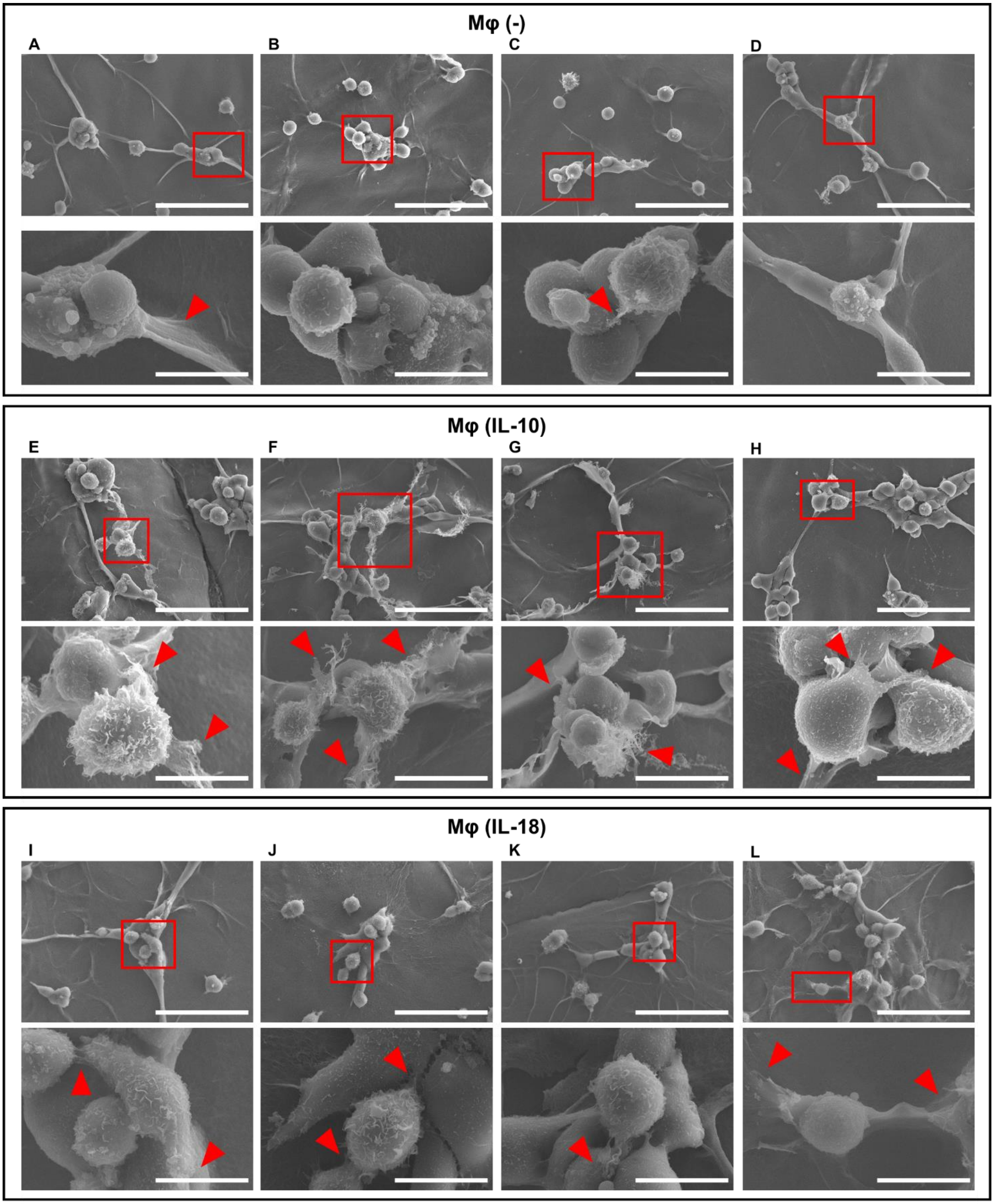

133

Supplementary Figure 8. **Ultrastructural Analysis of Cell-Cell Interaction between Several Subsets of Macrophages (Mφs) and Endothelia.** Ultrastructural analysis of cell-cell interaction between b.End5 cells and **(A-D)** Mφs (-), **(E-H)** Mφs [interleukin (IL)-10], or **(I-L)** Mφs (IL-18). All of SEM images were obtained at 4 h after co-culture of b.End5 cells with each subset of Mφs on Matrigel at 37°C under 5% CO_2. Lower images are magnified regions from red rectangles in the corresponding upper panels. Magnification and scale bars: **(A)** Upper; ×1.0 K, 50 µm, Lower; ×3.0 K, 10 µm, **(B)** Upper; ×1.0 K, 50 µm, Lower; ×5.0 K, 10 µm, **(C)** Upper; ×1.0 K, 50 µm, Lower; ×5.0 K, 10 µm, **(D)** Upper; ×1.0 K, 50 µm, Lower; ×3.0 K, 10 µm, **(E)** Upper; ×1.0 K, 50 µm, Lower; ×5.0 K, 10 µm, **(F)** Upper; ×1.0 K, 50 µm, Lower; ×3.0 K, 10 µm, **(G)** Upper; ×1.0 K, 50 µm, Lower; ×3.0 K, 10 µm, **(H)** Upper; ×1.0 K, 50 µm, Lower; ×5.0 K, 10 µm, **(I)** Upper; ×1.0 K, 50 µm, Lower; ×5.0 K, 10 µm, **(J)** Upper; ×1.0 K, 50 µm, Lower; ×5.0 K, 10 µm, **(K)** Upper; ×1.0 K, 50 µm, Lower; ×6.0 K, 10 µm, **(L)** Upper; ×1.0 K, 50 µm, Lower; ×5.0 K, 10 µm, respectively. Red arrowheads indicate pseudopodia of Mφs interacting with endothelial cells.

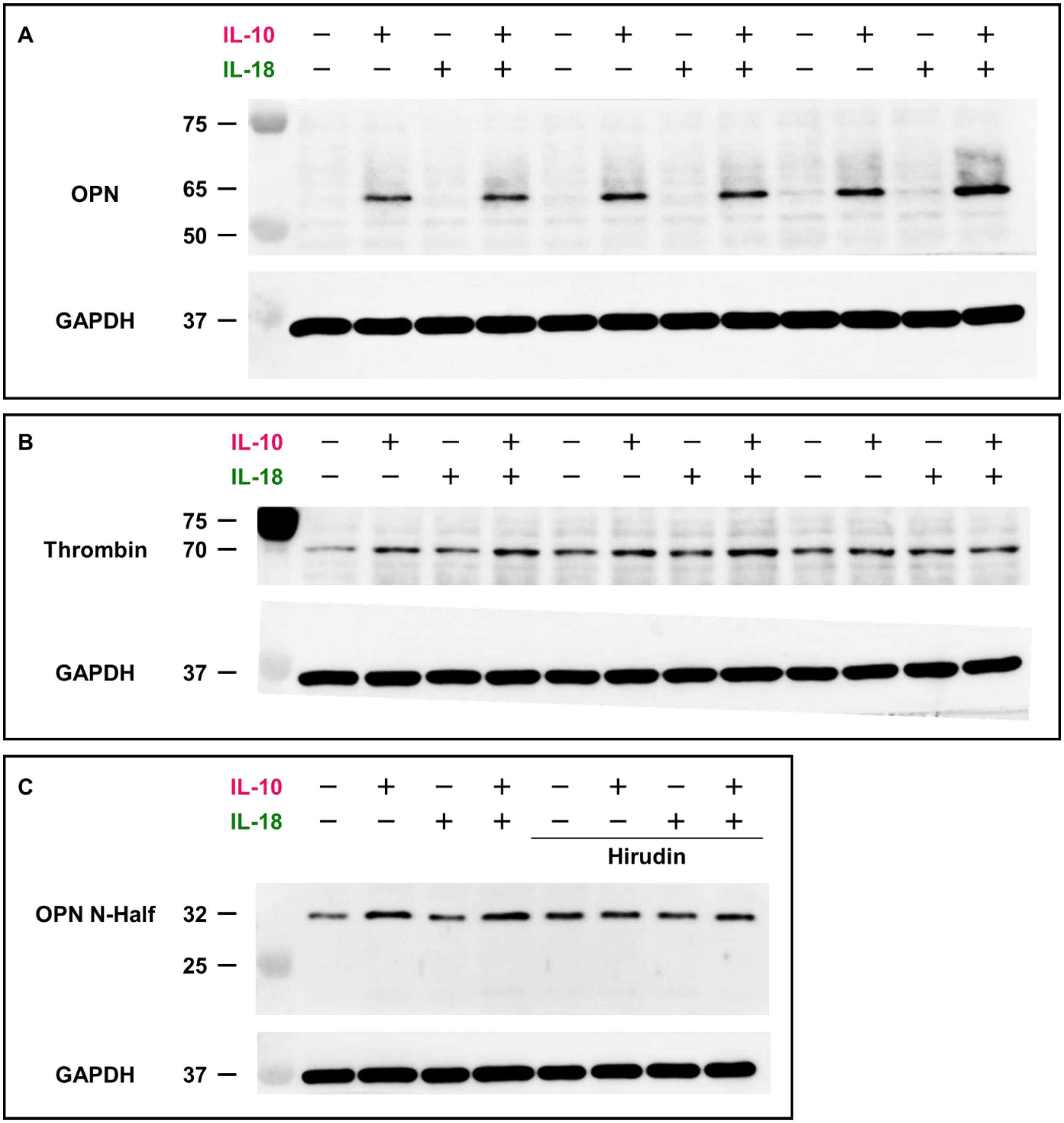

Supplementary Figure 9. **Original Western Blotting Images of Osteopontin (OPN), Thrombin, and OPN N-Half as well as Glyceraldehyde-3-Phosphate Dehydrogenase (GAPDH). (A)** The original western blotting images of OPN and GAPDH shown in Figure 5C. The observed bands size of OPN, GAPDH were approximately 65 or 37 kDa, respectively. **(B)** The original western blotting images of thrombin and GAPDH shown in Figure 6B. The observed bands size of thrombin, GAPDH were approximately 70 or 37 kDa, respectively. **(C)** The original western blotting images of OPN N-Half, reflecting thrombin-cleaved form of OPN, and GAPDH shown in Figure 6C. The observed bands size of OPN N-Half, GAPDH were approximately 32 or 37 kDa, respectively.

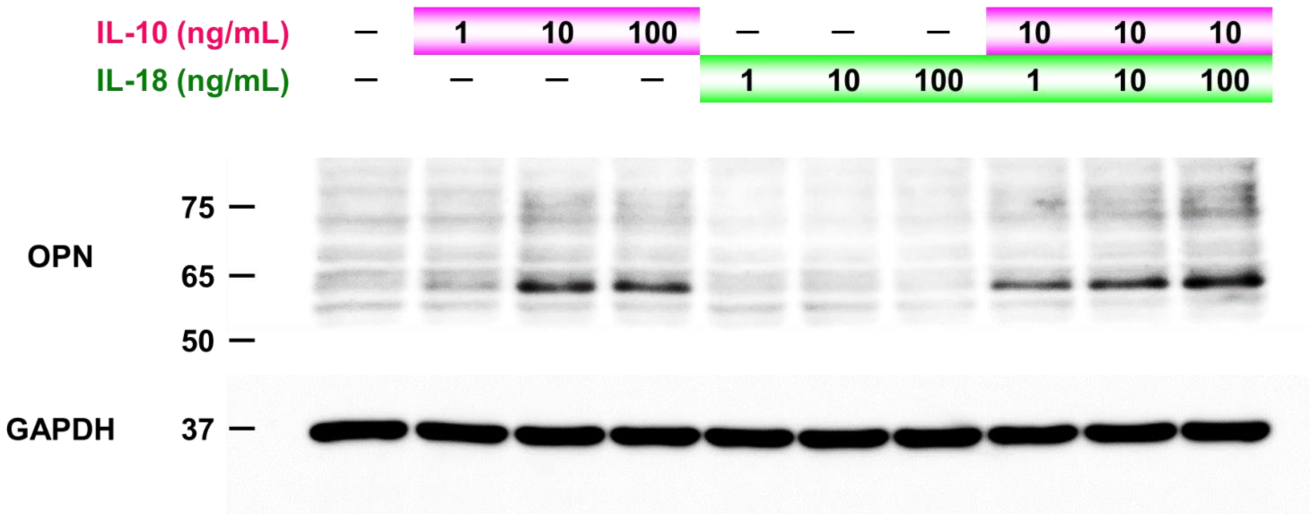

Supplementary Figure 10. Dose-dependent Increases in the Protein Expression Level of Osteopontin (OPN) in Macrophages (Mφs) Treated with Interleukin (IL)-10 and IL-18. After exposing RAW264.7 cells to either IL-10 (1-100 ng/mL) or IL-18 (1-100 ng/mL) alone or combination of IL-10 (10 ng/mL) with IL-18 (1-100 ng/mL) for 24 h at 37°C under 5% CO_2, the protein expression level of OPN in the whole cell fraction was measured by western blotting analysis. The upper and lower panels are the original western blotting images of OPN or glyceraldehyde-3-phosphate dehydrogenase (GAPDH), respectively. The observed bands size of OPN, GAPDH were approximately 65 or 37 kDa, respectively. Exposure of RAW264.7 cells to IL-10 at concentrations of 10 ng/mL and 100 ng/mL but not at 1 ng/mL almost equally increased the amount of OPN protein, whereas IL-18 at any concentration range was not sufficient for up-regulation of OPN level. Conversely, the concomitant use of IL-18 dose-dependently amplified an increase in OPN expression level elicited by IL-10 (10 ng/mL) alone.

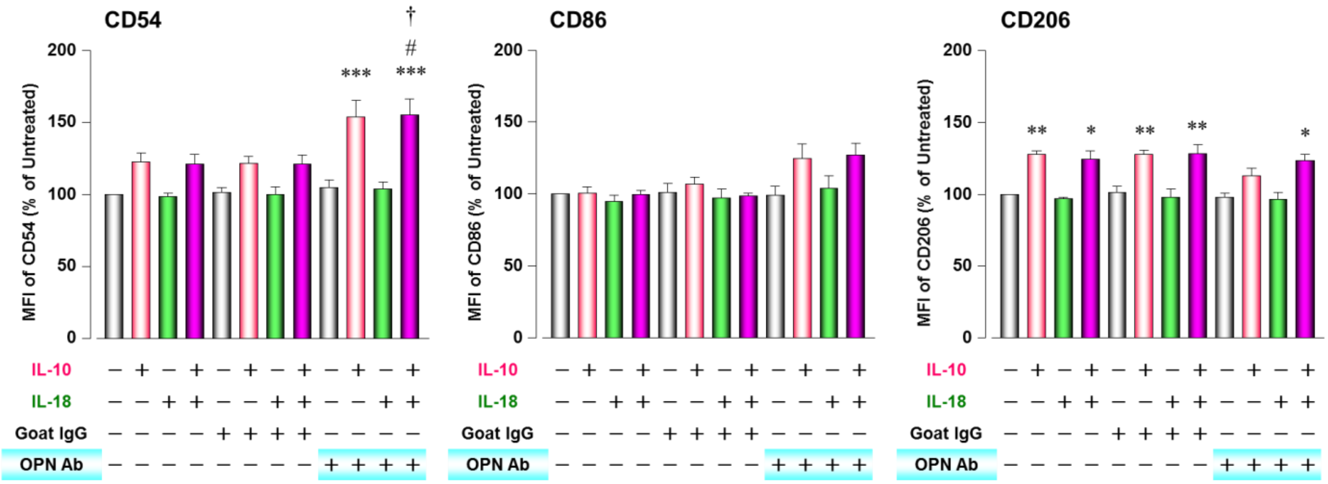

Supplementary Figure 11. **Influence of Neutralizing Antibody against Osteopontin (OPN) on the Surface Expressions of CD54, CD86, and CD206.** After incubating RAW264.7 cells with interleukin (IL)-10 (10 ng/mL) or IL-18 (100 ng/mL) alone or their combination use in the presence or absence of a neutralizing Ab against OPN (3 µg/mL) or its isotype-matched control Ab (3 µg/mL) for 24 h at 37°C under 5% CO_2, the mean fluorescence intensities (MFIs) of CD54, CD86, and CD206 on the surface membrane were measured by FACS analysis. Treatment of RAW264.7 cells with IL-10 (10 ng/mL) alone or that combined with IL-18 (100 ng/mL) slightly increased the surface expression of CD54, which was significantly augmented by concomitant treatment with an anti-OPN Ab. This Ab slightly increased the surface level of CD86 only when IL-10 (10 ng/mL) with or without IL-18 (100 ng/mL) was present, unaccompanied by any impact on the significant increase in the surface expression of CD206. n = 3-4. Data are expressed as means ± SEM and were analyzed by a one-way ANOVA followed by Tukey's test. $^{***}p < 0.001$, $^{**}p < 0.01$, $^{*}p < 0.05$ vs. untreated, $^{\#}p < 0.05$ vs. IL-10 alone, $^{\dagger}p < 0.05$ vs. IL-10 + IL-18.

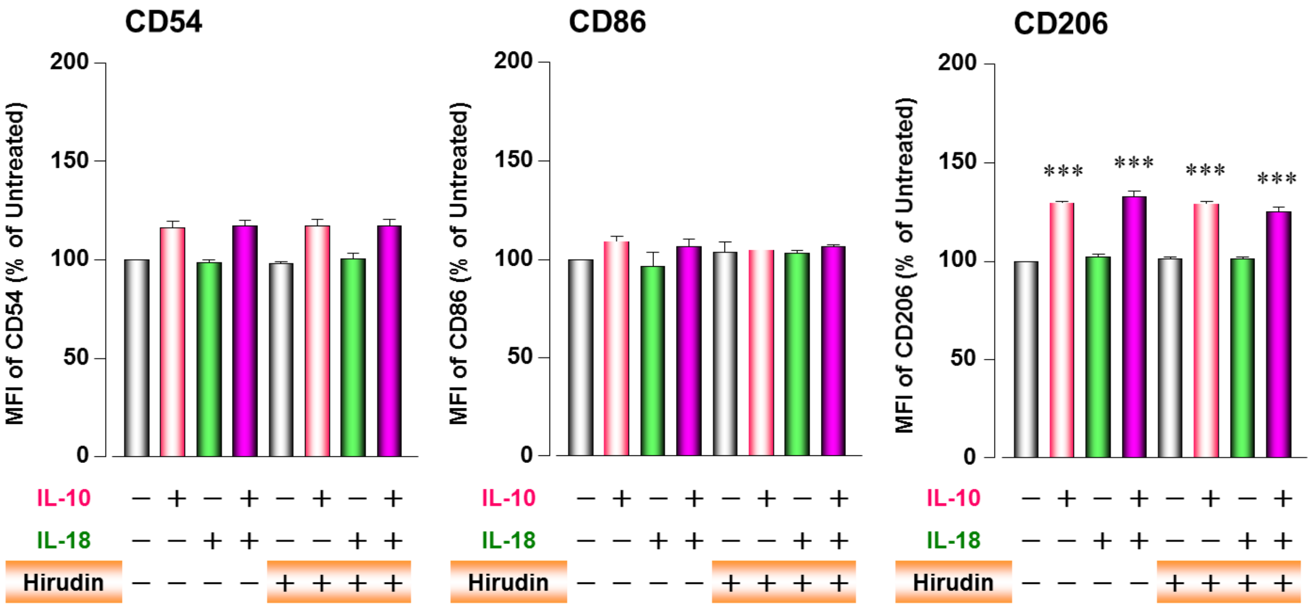

Supplementary Figure 12. Influence of a Pharmacological Inhibitor for Thrombin on the Surface Expressions of CD54, CD86, and CD206. After incubating RAW264.7 cells with interleukin (IL)-10 (10 ng/mL) or IL-18 (100 ng/mL) alone or their combination use in the presence or absence of hirudin (1 µg/mL) for 24 h at 37°C under 5% CO_2, the mean fluorescence intensities (MFIs) of CD54, CD86, and CD206 on the surface membrane were determined by FACS analysis. Treatment with hirudin (1 µg/mL), a specific thrombin inhibitor, had little impact on CD54, CD86, and CD206 expression on the surface of RAW264.7 cells exposed to either IL-10 (10 ng/mL) or IL-18 (100 ng/mL) alone or their combination use. n = 3-4. Data are expressed as means ± SEM and were analyzed by a one-way ANOVA followed by Tukey's test. $^{***}p < 0.001$ vs. untreated.

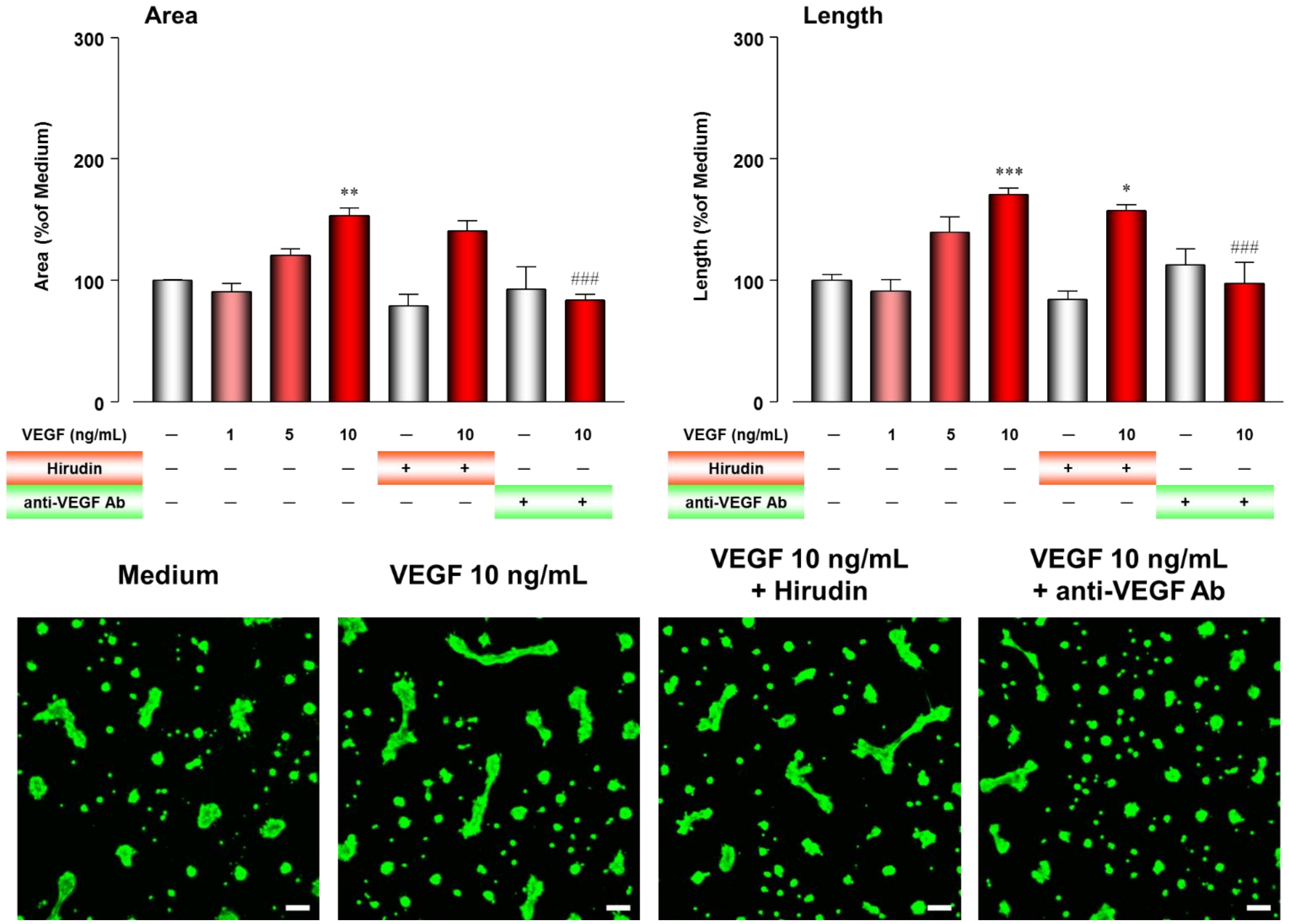

Supplementary Figure 13. **Influence of a Pharmacological Inhibitor for Thrombin on Vascular Endothelial Growth Factor (VEGF)-dependent Angiogenic Induction.** The b.End5 cells were seeded on Matrigel, and subsequently incubated for 16 h at 37°C under 5% CO_2 in the medium containing VEGF (1, 5, or 10 ng/mL) in the presence or absence of an anti-VEGF antibody (Ab) (10 µg/mL) or a specific inhibitor for thrombin, hirudin (1 µg/mL). Stimulation of b.End5 cells by VEGF gradually promoted the tube formation in a dose-dependent manner. Significant increase in the degree of tube-like network induced by VEGF 10 ng/mL was not abolished by concomitant treatment with hirudin, but was dramatically inhibited by an anti-VEGF Ab to the level observed in the medium only group. n = 4. Data are expressed as means ± SEM and were analyzed by a one-way ANOVA followed by Tukey's test. $^{***}p < 0.001$, $^{**}p < 0.01$, $^{*}p < 0.05$ vs. medium only group, $^{\#\#\#}p < 0.001$ vs. VEGF 10 ng/mL alone. Lower panels are representative pictures of tube-like structures visualized by calcein acetoxymethylester staining. Scale bar represents 100 µm.

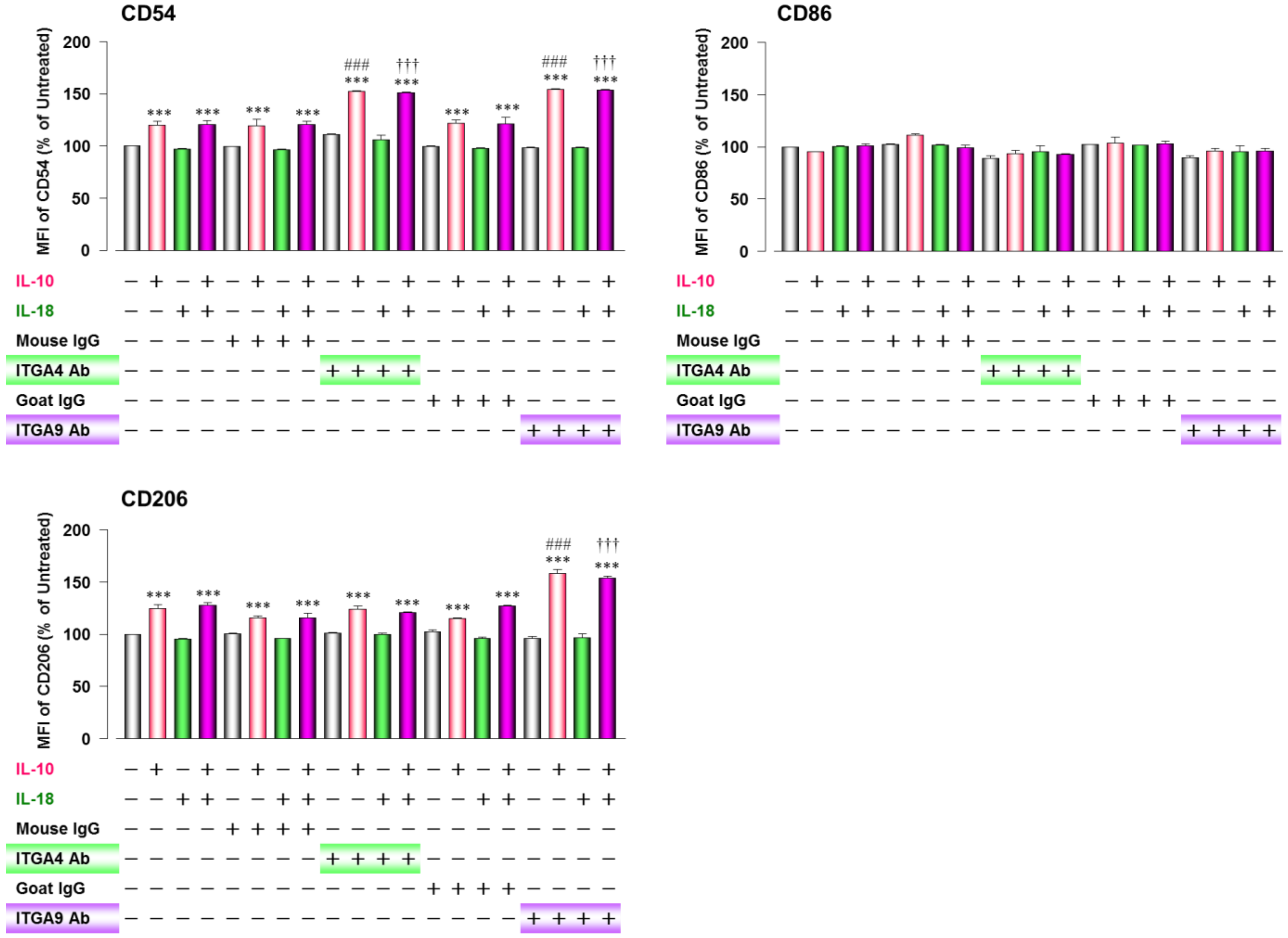

Supplementary Figure 14. Influence of Blocking Antibodies (Abs) against Integrins α4/α9 on the Surface Expressions of CD54, CD86, and CD206. After incubating RAW264.7 cells with interleukin (IL)-10 (10 ng/mL) or IL-18 (100 ng/mL) alone or their combination use in the presence or absence of a neutralizing Ab against integrin α4 (10 μg/mL) or that against integrin α9 (10 μg/mL), or each isotype-matched control Ab (10 μg/mL) for 24 h at 37°C under 5% CO_2, the mean fluorescence intensities (MFIs) of CD54, CD86, and CD206 on the surface membrane were measured by FACS analysis. A neutralizing Ab against integrin α9 but not α4 significantly enhanced the increase in CD206 expression on the surface of RAW264.7 cells induced by IL-10 (10 ng/mL) with or without IL-18 (100 ng/mL). Moreover, both Abs given in combination with IL-10 (10 ng/mL) in the presence or absence of IL-18 (100 ng/mL) significantly amplified an increase in the expression of CD54 on the surface of RAW264.7 cells. These Abs had no effect on CD86 expression. n = 3-4. All data are expressed as means ± SEM and were analyzed by a one-way ANOVA followed by Tukey's test. $^{***}p < 0.001$ vs. untreated, $^{\#\#\#}p < 0.001$ vs. IL-10 alone, $^{\dagger\dagger\dagger}p < 0.001$ vs. IL-10 + IL-18. Integrin α4 = ITGA4; Integrin α9 = ITGA9.

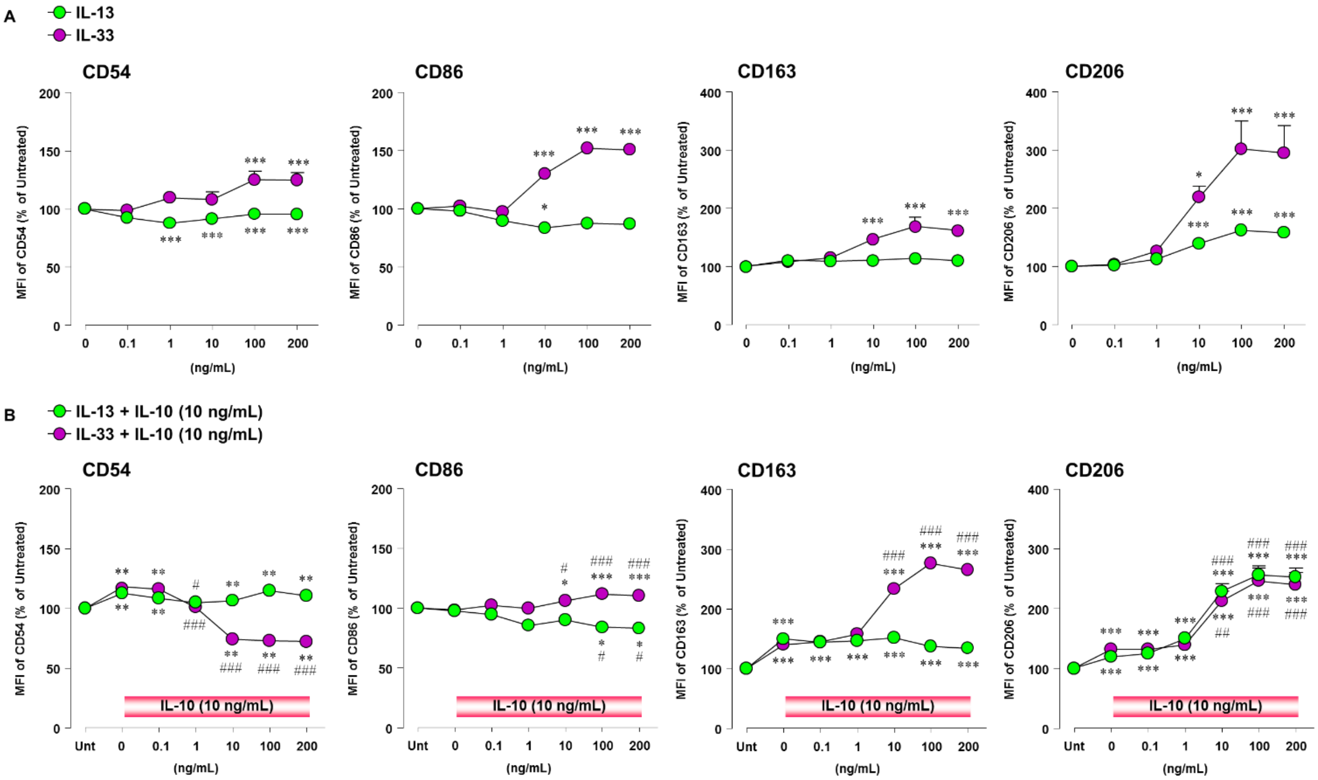

Supplementary Figure 15. **Effects of Interleukin (IL)-13 and IL-33 Stimuli on the Surface Expression Levels of Macrophage (Mφ) M1/M2 Markers. (A)** After incubating RAW264.7 cells with either IL-13 (0.1 – 200 ng/mL) or IL-33 (0.1 – 200 ng/mL) alone for 24 h at 37°C under 5% CO_2, the mean fluorescent intensities (MFIs) of CD54, CD86, CD163, and CD206 on the surface membrane were determined by FACS analysis. Treatment with IL-33 at the concentration ranging from 0.1 to 200 ng/mL markedly increased the surface levels of CD86, CD163, and CD206 in a dose-dependent manner, while that of CD54 was moderately increased in comparison with untreated cells. Treatment with IL-13 increased the surface level of CD206 in a dose-dependent manner, while there were no significant changes in the surface levels of CD54, CD86, and CD163 at the concentration ranging from 0.1 to 200 ng/mL. n = 4. Data are expressed as means ± SEM and were analyzed by a one-way ANOVA followed by Dunnet's test. $^{***}p < 0.001$, $^{*}p < 0.05$ vs. untreated. **(B)** After incubating RAW264.7 cells with either IL-13 (0.1 – 200 ng/mL) or IL-33 (0.1 – 200 ng/mL) in combination with IL-10 (10 ng/mL) for 24 h at 37°C under 5% CO_2, the MFIs of CD54, CD86, CD163, and CD206 on the surface membrane were determined by FACS analysis. Treatment of IL-33 in combination with IL-10 significantly decreased the expression level of CD54, although IL-33 alone increased that of CD54 with little influence on that of CD86. In contrast, IL-33 is not sufficient for amplifying increases in CD163 and CD206 levels induced by IL-10, whereas IL-33 alone increased CD163 and CD206 levels in a concentration-dependent manner. Concomitant treatment with IL-13 and IL-10 had little effects on changes in CD54, CD86, and CD163 levels induced by IL-10. By contrast, IL-13 dose-dependently and markedly amplified an increase in CD206 level induced

by IL-10, although IL-13 alone also increased CD206 moderately. n = 4. Data are expressed as means ± SEM and were analyzed by a one-way ANOVA followed by Tukey's test. $^{***}p < 0.001$, $^{**}p < 0.01$, $^{*}p < 0.05$ vs. untreated, $^{###}p < 0.001$, $^{##}p < 0.01$, $^{#}p < 0.05$ vs. IL-10 alone.

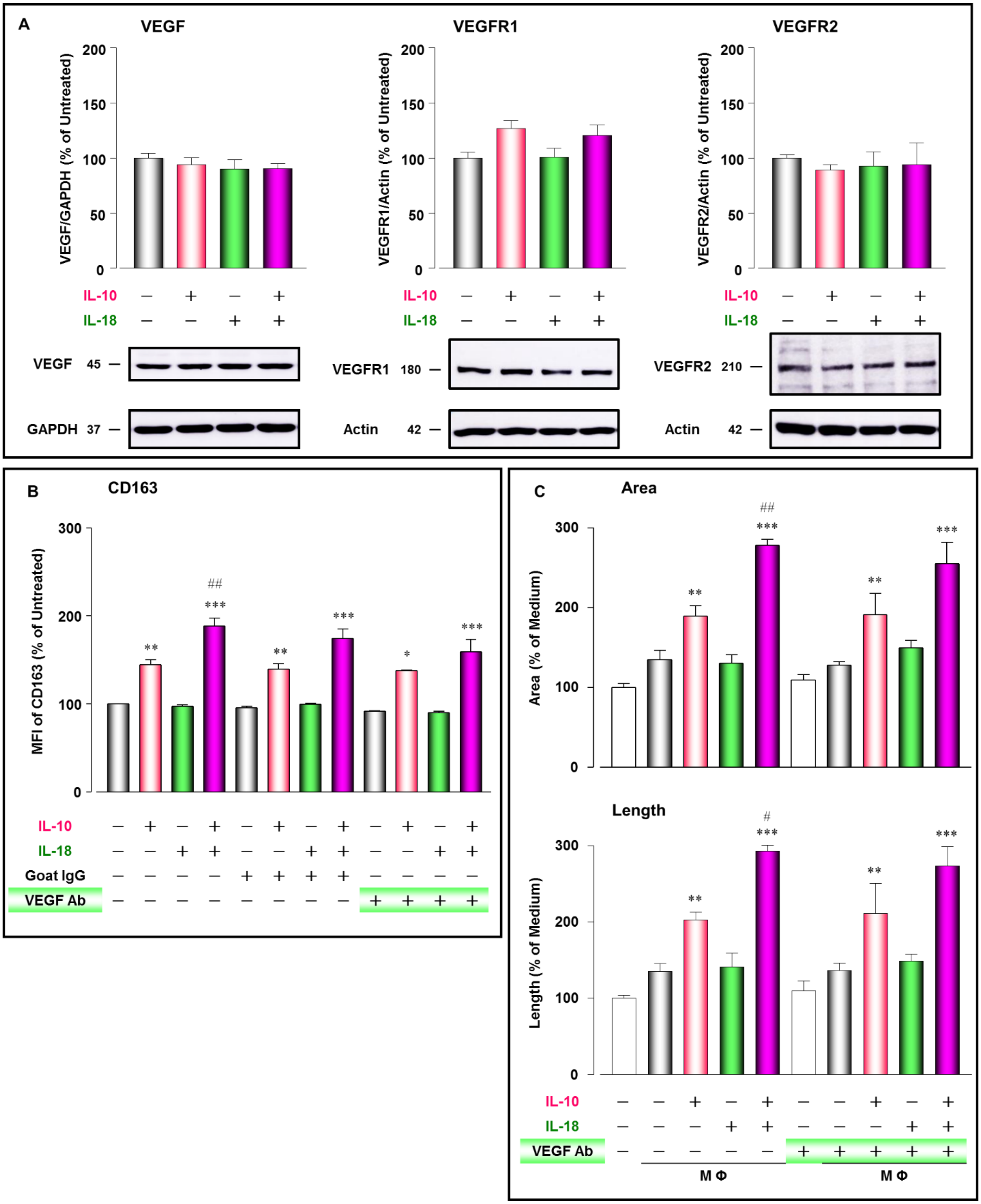
A
VEGF
VEGFR1
VEGFR2
VEGF/GAPDH (% of Untreated)
VEGFR1/Actin (% of Untreated)
VEGFR2/Actin (% of Untreated)
IL-10
IL-18
VEGF 45
GAPDH 37
VEGFR1 180
Actin 42
VEGFR2 210
Actin 42
B
CD163
MFI of CD163 (% of Untreated)
IL-10
IL-18
Goat IgG
VEGF Ab
C
Area
Area (% of Medium)
Length
Length (% of Medium)
IL-10
IL-18
VEGF Ab
M Φ
M Φ

Supplementary Figure 16. **Involvement of Vascular Endothelial Growth Factor (VEGF) in the Upregulation of CD163 Expression Levels and Angiogenic Capacity of M2-like Macrophages (Mφs). (A)** The protein expression levels of VEGF, VEGF receptor (R)1 and VEGFR2 were measured by western blotting analysis using whole cell lysates of RAW264.7 cells exposed to either interleukin (IL)-10 (10 ng/mL) and IL-18 (100 ng/mL) alone or in combination for 24 h at 37°C under 5% CO_2. Exposure of RAW264.7 cells to IL-10 (10 ng/mL) or IL-18 (100 ng/mL) alone or their combination had little impact on the protein expression levels of VEGF, a well-characterized angiogenic mediator as well as VEGFR1 and VEGFR2, both of which are receptors for VEGF. Relative levels of VEGF, VEGFR1, or VEGFR2 are shown as a ratio of (left) VEGF/glyceraldehyde-3-phosphate dehydrogenase (GAPDH), (middle) VEGFR1/actin, or (right) VEGFR2/actin. Lower panels are typical images of each protein. n = 6-9. **(B)** After incubating RAW264.7 cells with either IL-10 (10 ng/mL) or IL-18 (100 ng/mL) alone or their combination use in the presence or absence of a neutralizing antibody (Ab) against VEGF (10 µg/mL) or its isotype-matched control Ab (10 µg/mL) for 24 h at 37°C under 5% CO_2, relative mean fluorescence intensity (MFI) of CD163 on the surface membrane were measured by FACS analysis. An anti-VEGF Ab never had significant influences on increases in the surface expression of CD163 observed in Mφ (IL-10) and Mφ (IL-10 + IL-18). n = 3. Data are expressed as means ± SEM and were analyzed by a one-way ANOVA followed by Tukey's test. $^{***}p < 0.001$, $^{**}p < 0.01$, $^{*}p < 0.05$ vs. untreated, $^{\#\#}p < 0.01$ vs. IL-10 alone. **(C)** After polarizing RAW264.7 cells with either IL-10 (10 ng/mL) or IL-18 (100 ng/mL) alone or their combination use for 24 h at 37°C under 5% CO_2, they were co-cultured with b.End5 cells in the presence or absence of a neutralizing Ab against VEGF (10 µg/mL) on the Matrigel for 16 h at 37°C under 5% CO_2. The total areas and lengths of tube-like structures were determined by the Matrigel tube formation assay. There were no significant impacts on increases in the area and length of tube-like structures in Mφ (IL-10) and Mφ (IL-10 + IL-18) by treatment with an anti-VEGF Ab. n = 6. $^{***}p < 0.001$, $^{**}p < 0.01$ vs. untreated, $^{\#\#}p < 0.01$, $^{\#}p < 0.05$ vs. IL-10 alone. All data are expressed as means ± SEM and were analyzed by a one-way ANOVA followed by Tukey's test.

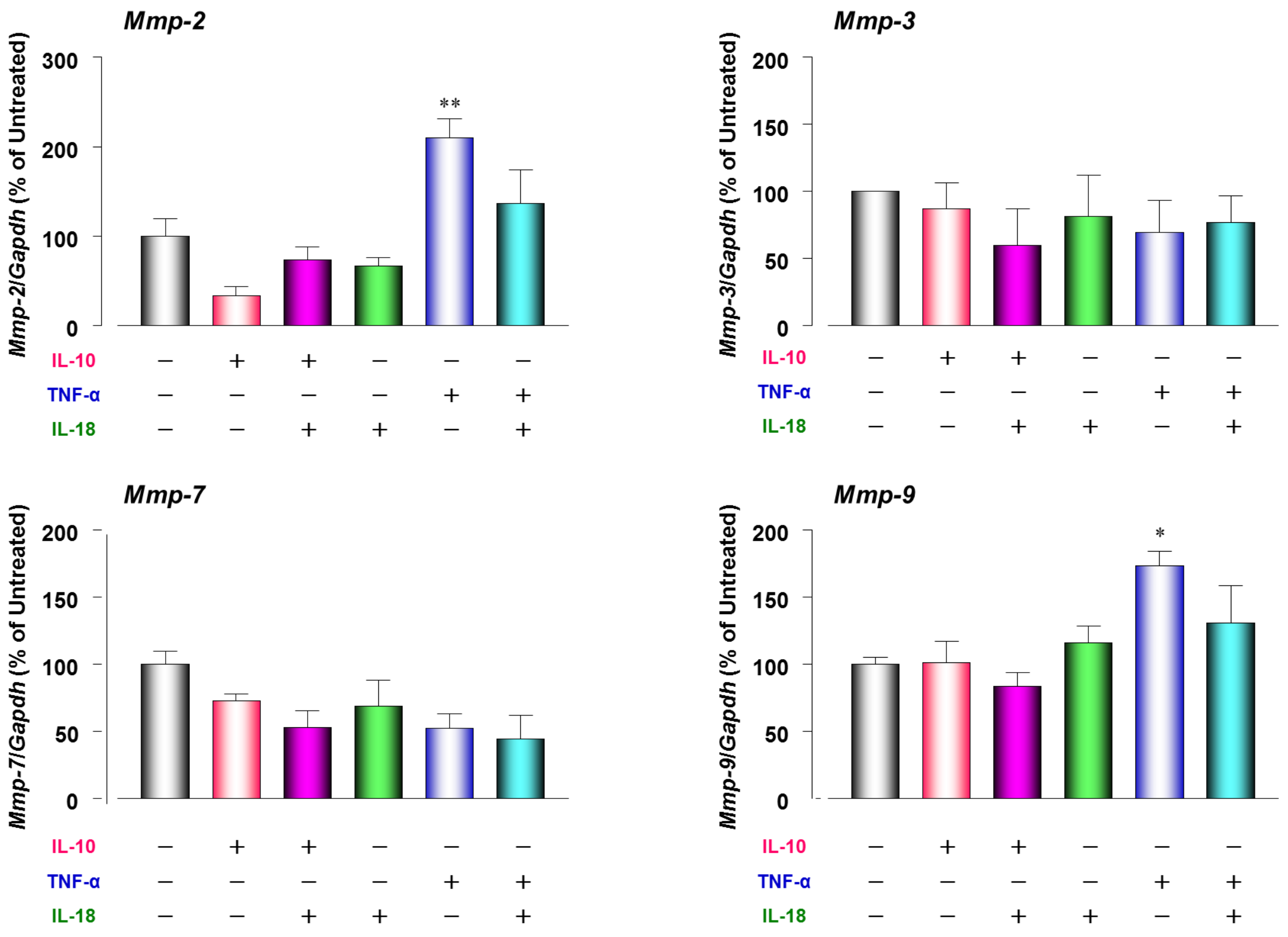

Supplementary Figure 17. **Changes in the Expression Levels of Matrix Metalloproteinases (MMPs) in Several Subsets of Macrophages (Mφs).** After incubating RAW264.7 cells with interleukin (IL)-10 (10 ng/mL), tumor necrosis factor (TNF)-α (5 ng/mL), or IL-18 (100 ng/mL) each alone or in combination for 24 h at 37°C under 5% CO_2, the mRNA levels of MMPs, such as *Mmp-2*, *-3*, *-7*, and *-9* were analyzed by the real-time reverse transcription polymerase chain reaction. Exposure of RAW264.7 cells to TNF-α (5 ng/mL) dramatically increased the mRNA expression of *Mmp-2* and *Mmp-9*. These increases were suppressed moderately by concomitant treatment with IL-18 (100 ng/mL), without any alterations in the mRNA expression of *Mmp-3* and *Mmp-7*. By contrast, IL-10 (10 ng/mL) or IL-18 (100 ng/mL) alone or in combination had no significant impact on the mRNA expression of the four MMPs measured in this study. Relative levels of *Mmp-2*, *Mmp-3*, *Mmp-7*, or *Mmp-9* are shown as a ratio of (upper left) *Mmp-2*/*glyceraldehyde-3-phosphate dehydrogenase* (*Gaphd*), (upper right) *Mmp-3*/*Gaphd*, (lower left) *Mmp-7*/*Gaphd*, or (lower right) *Mmp-9*/*Gaphd*, respectively. n = 4-7. All data are expressed as means ± SEM and were analyzed by a one-way ANOVA followed by Tukey's test. $^{**}p < 0.01$, $^{*}p < 0.05$ vs. untreated.

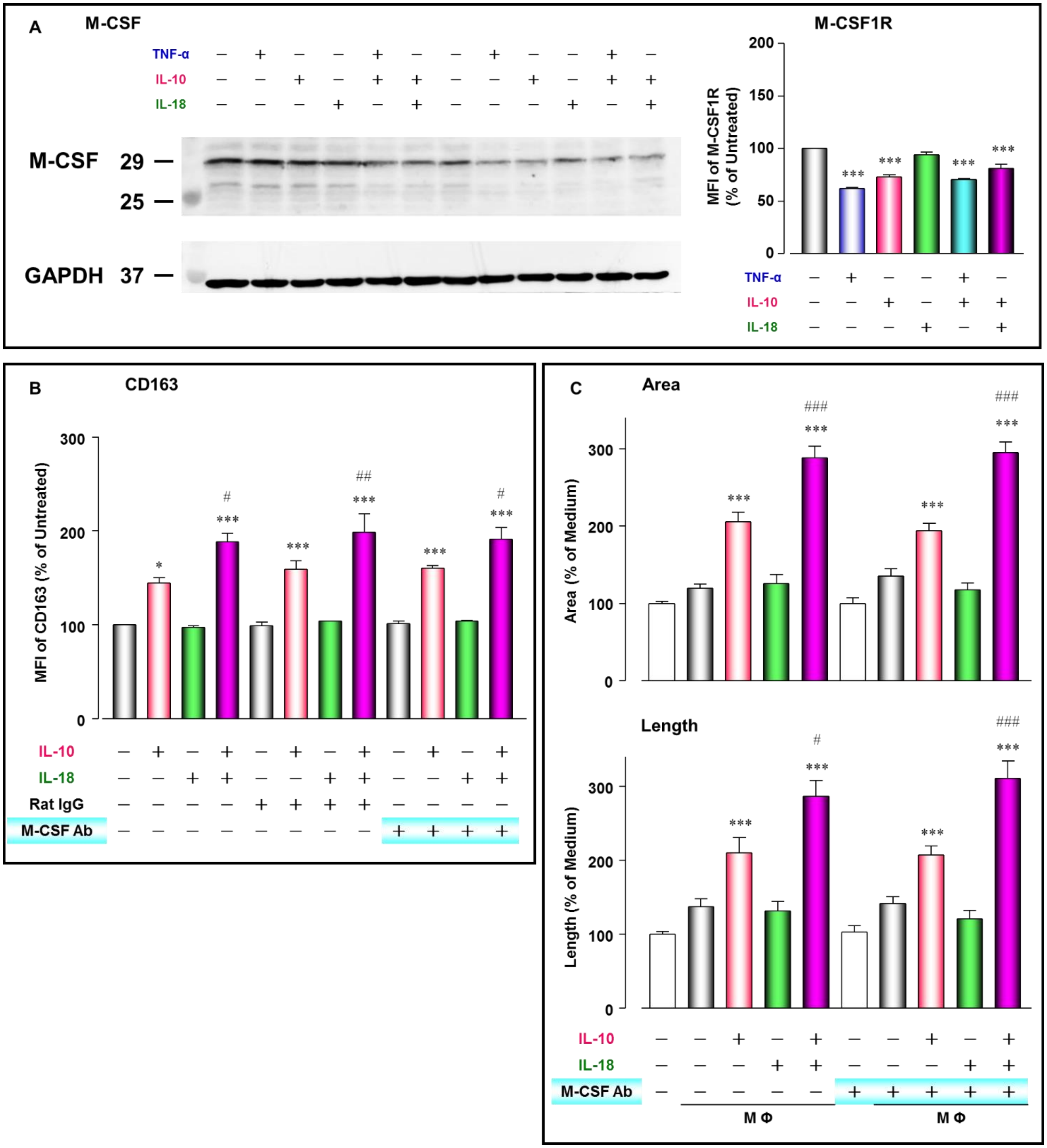
A
M-CSF
TNF-α
IL-10
IL-18
M-CSF 29
25
GAPDH 37
M-CSF1R
MFI of M-CSF1R (% of Untreated)
B
CD163
MFI of CD163 (% of Untreated)
Rat IgG
M-CSF Ab
C
Area
Area (% of Medium)
Length
Length (% of Medium)
M Φ

295

Supplementary Figure 18. **Involvement of Macrophage (Mφ) Colony-Stimulating Factor (M-CSF) in the Enhancements in CD163 Expression Levels and Angiogenic Capacity of M2-like Mφs. (A)** After incubating RAW264.7 cells with either tumor necrosis factor (TNF)-α (5 ng/mL) and interleukin (IL)-10 (10 ng/mL) alone or in combination with IL-18 (100 ng/mL) for 24 h at 37°C under 5% CO_2, the protein expression of M-CSF in the whole cellular fraction or the mean fluorescent intensity (MFI) of the cell surface expression of M-CSF1 receptor (M-CSFR) were determined by western blotting analysis or FACS analysis, respectively. Treatment with either TNF-α (5 ng/mL) or IL-10 (10 ng/mL) alone or in combination with IL-18 (100 ng/mL) almost equally decreased the protein expression of M-CSF and M-CSF1R in comparison with untreated group. Left panel shows the typical western blotting images of M-CSF and glyceraldehyde-3-phosphate dehydrogenase (GAPDH). Right column indicates the relative MFI of M-CSF1R. n = 4. Data are expressed as means ± SEM and were analyzed by a one-way ANOVA followed by Tukey's test. $^{***}p < 0.001$ vs. untreated. **(B)** After incubating RAW264.7 cells with either IL-10 (10 ng/mL) or IL-18 (100 ng/mL) alone or their combination use in the presence or absence of a neutralizing antibody (Ab) against M-CSF (50 ng/mL) or its isotype-matched control Ab (50 ng/mL) for 24 h at 37°C under 5% CO_2, the relative MFI of CD163 on the surface membrane were measured by FACS analysis. An anti-M-CSF Ab had little impacts on increases in the surface expression of CD163 observed in Mφ (IL-10) and Mφ (IL-10 + IL-18). n = 3. Data are expressed as means ± SEM and were analyzed by a one-way ANOVA followed by Tukey's test. $^{***}p < 0.001$, $^{*}p < 0.05$ vs. untreated, $^{##}p < 0.01$, $^{#}p < 0.05$ vs. IL-10 alone. **(C)** After polarizing RAW264.7 cells with either IL-10 (10 ng/mL) or IL-18 (100 ng/mL) alone or their combination use for 24 h at 37°C under 5% CO_2, they were co-cultured with b.End5 cells in the presence or absence of a neutralizing Ab against M-CSF (50 ng/mL) on the Matrigel for 16 h at 37°C under 5% CO_2. The total areas and lengths of tube-like structures were determined by the Matrigel tube formation assay. Treatment with an anti-M-CSF Ab showed little influences on increases in the area and length of tube-like structures observed in Mφ (IL-10) and Mφ (IL-10 + IL-18). n = 8. $^{***}p < 0.001$ vs. untreated, $^{###}p < 0.001$, $^{#}p < 0.05$ vs. IL-10 alone. All data are expressed as means ± SEM and were analyzed by a one-way ANOVA followed by Tukey's test.

Supplementary Videos

Supplementary Video 1. Temporal Behavior of Macrophages (Mφs) [interleukin (IL)-10 + IL-18] in the Time-lapse Live-cell Imaging of Matrigel Tube Formation Assay. PKH67 (green)-labeled b.End5 cells (6.0 × 10^5 cells) were co-cultured with PKH26 (red)-labeled Mφs (IL-10 + IL-18) (1.5 × 10^5 cells) on the Matrigel at 37°C under 5% CO_2 conditions. Time-lapse live-cell imaging in the Matrigel tube formation was obtained every 10 minutes over 16 h. After starting observation, PKH67 (green)-labeled b.End5 cells were in contact with each other from far and wide. Subsequently, the cell populations connected to each other through the help of PKH26 (red)-labeled-Mφ (IL-10 + IL-18) from 3 h to 8 h, driving rapid induction of angiogenesis that was almost completed until 12 h and reached a plateau phase toward the end of observation period.

Supplementary Videos 2-4. Temporal Behavior of Several Macrophages (Mφs) Subsets in the Time-lapse Live-cell Imaging of Matrigel Tube Formation Assay. PKH67 (green)-labeled b.End5 cells (6.0 × 10^5 cells) were co-cultured with 1.5 × 10^5 cells of PKH26 (red)-labeled Mφs (-) (**Video S2**), Mφs [interleukin (IL)-10] (**Video S3**), or Mφs (IL-18) (**Video S4**) on the Matrigel at 37°C under 5% CO_2 conditions. Time-lapse live-cell imaging in the Matrigel tube formation was obtained every 10 minutes over 16 h. After starting observation, PKH67 (green)-labeled b.End5 cells were in contact with each other from far and wide. Subsequently, the cell populations connected to each other through the help of PKH26 (red)-labeled-Mφ (IL-10) but not of Mφ (-) and Mφ (IL-18) from 3 h to 8 h, driving rapid induction of angiogenesis that was almost completed until 12 h and reached a plateau phase toward the end of observation period.

Supplementary Video 5. Characteristic Behavior of Macrophages (Mφs) [interleukin (IL)-10 + IL-18] during *in vitro* Angiogenesis Induction. The **Video S5** was reconstructed from a series of high magnification images in a selected field of Video S1. Apart of PKH26 (red)-labeled Mφ (IL-10 + IL-18) spread pseudopodia wide apart, hereby captured and brought PKH67 (green)-labeled endothelial cells into close apposition of vascular tube. Subsequently, this Mφ attains supportive role to keep endothelium at capillaries where vascular sprouting and/or junction occur probably by bridging between endothelial cells, which seemed to be a driving force for angiogenesis. In addition, this Mφ remained in contact with vessels for at least some time after vascular tubes had fused to form a vascular intersection albeit moving to another parts of vascular tube network.
